# Synthesis of 9-Hydroxystearic Acid Derivatives and Their Antiproliferative Activity on HT 29 Cancer Cells

**DOI:** 10.3390/molecules24203714

**Published:** 2019-10-15

**Authors:** Natalia Calonghi, Carla Boga, Dario Telese, Silvia Bordoni, Giorgio Sartor, Chiara Torsello, Gabriele Micheletti

**Affiliations:** 1 Department of Pharmacy and Biotechnology, University of Bologna, 40127 Bologna, Italy; giorgio.sartor@unibo.it (G.S.); chiara.torsello@virgilio.it (C.T.); 2 Department of Industrial Chemistry ‘Toso Montanari’, Alma Mater Studiorum Università di Bologna Viale Del Risorgimento 4, 402136 Bologna, Italy; dario.telese2@unibo.it (D.T.); silvia.bordoni@unibo.it (S.B.); gabriele.micheletti3@unibo.it (G.M.)

**Keywords:** 9-hydroxystearic acid, methyl 9-hydroxystearate, methyl 9-aminostearate, cancer

## Abstract

9-Hydroxystearic acid (9-HSA) is an endogenous cellular lipid that possesses antiproliferative and selective effects against cancer cells. A series of derivatives were synthesized in order to investigate the effect of the substituent in position 9 and on the methyl ester functionality on the biological activity. The two separate enantiomers of methyl 9-hydroxystearate and of methyl 9-aminostearate showed antiproliferative activity against the HT29 cell line. This indicates the importance of position 9 groups being able to make hydrogen bonding with the molecular target. Further, this effect must be preserved when the carboxy group of 9-HSA is esterified. The biological tests showed that the amines, contrarily to methyl esters, resulted in cytotoxicity. A deep investigation on the effect of methyl (*R*)-9-hydroxystearate on HT29 cells showed an antiproliferative effect acting through the CDKN1A and MYCBP gene expression.

## 1. Introduction

**Figure 1:**
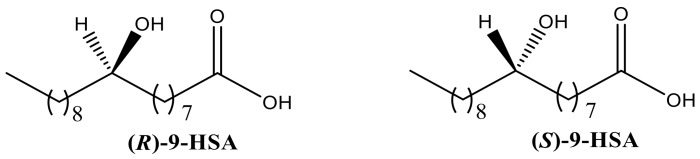
The two enantiomeric forms of 9-hydroxystearic acid, an HDAC inhibitor.

9-Hydroxystearic acid (9-HSA, Figure 1) is an endogenous cellular lipid that possesses a natural negative regulatory activity of tumor cell proliferation. [1,2,3] Its content has been found greatly diminished in cancer cells with respect to the corresponding normal line [1] and its exogenous administration to adenocarcinoma (HT29), [4] osteosarcoma (U2OS, [5] or SaOS [6]) cancer cell lines results in a significant inhibition of the proliferation rate, as well as a significant increase of p53-independent p21 expression. [4] The expression of the cell cycle kinase inhibitor CDKN1A (P21) is induced in neoplastic cells by inhibitors of histone deacetylase 1 (HDAC1). Actually, the authors found that 9-HSA acts as a competitive inhibitor of the HDAC1 (histone deacetylase 1) isoform [7,8]. The studies of molecular docking [7] showed an interaction between the 9-HSA carboxy group and the zinc ion in the active site of HDAC1, by a mechanism similar to that already known for HDAC inhibitors such as valproate, butyrate, trichostatin A (TSA), and suberoylanilidehydroxamic acid (SAHA, Vorinostat) [9]. The molecular docking also predicted a more favorable binding energy for the HDAC1-(*R*)-9-HSA complex, with respect to the complex formed with the opposite enantiomer (*S*)-9-HSA. Enantiopure (*R*)-9-HSA is more easily accessible within the natural chiral pool since its precursor, (*S*)-dimorphecholic acid, is one of the main components of seeds of the genus Dimorphotheca plants [10]. Thus, both the (*R*)-9-HSA and its enantiomer were synthesized, and the docking prediction experimentally confirmed [11]. Based on the above, the importance of both the carboxy- and the hydroxyl- group in inducing the antiproliferative activity of 9-HSA is apparent.

Hence, this study made some modifications on these key positions to better enlighten the role played by these functionalities and to design, if possible, some structure-activity relationships. Herein, the results obtained are reported.

## 2. Results and Discussion

### 2.1. Synthesis of 9-HSA Derivatives

**Figure 2:**
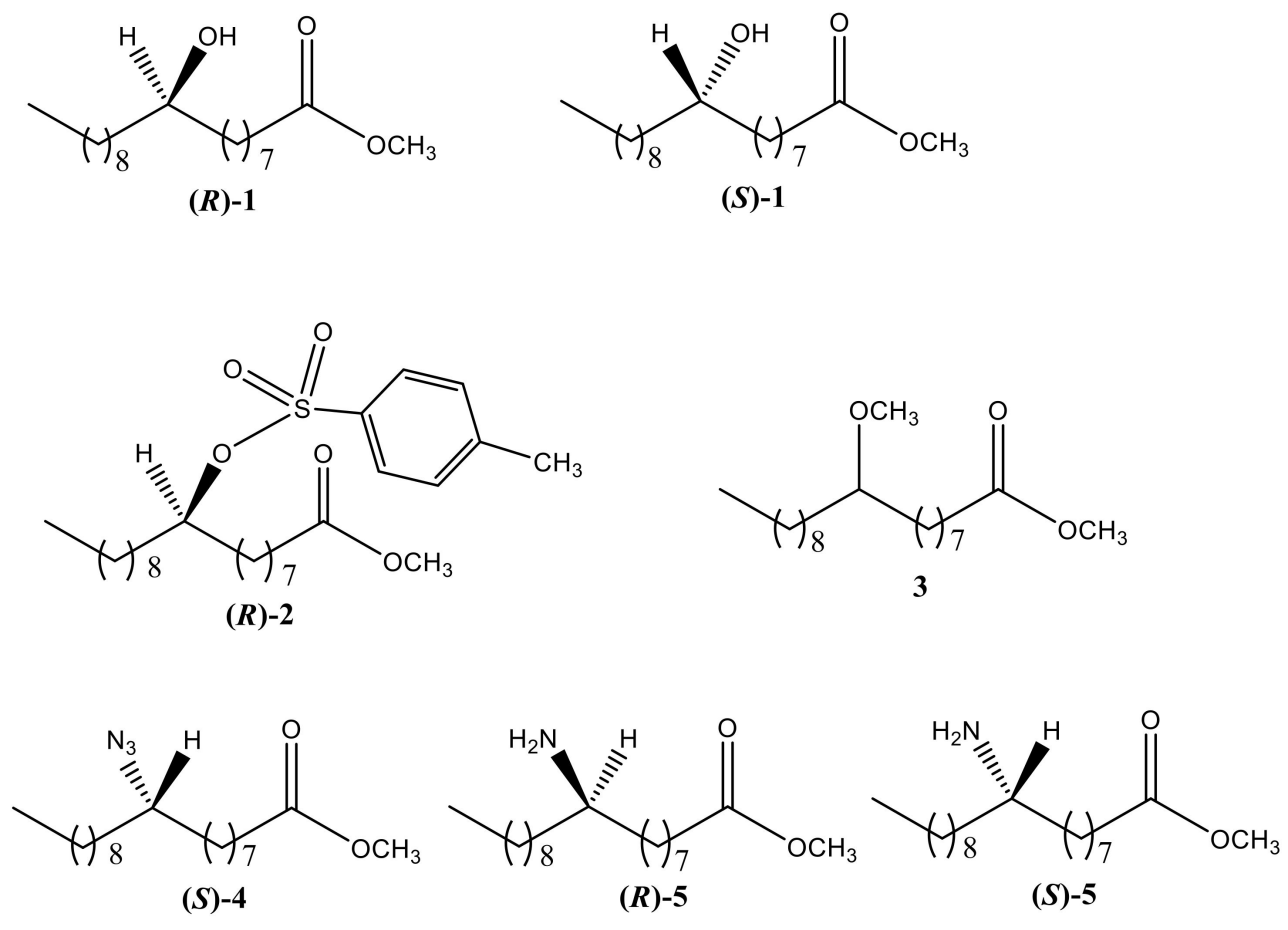
9-HSA derivatives synthesized and biologically tested.

The 9-HSA derivatives synthesized and investigated are in Figure 2.

Compounds **(*R*)**-**2** and **3** bear an ester and an ether group bound to the C-9, respectively, and this difference might give information on the free hydroxyl functionality about the biological action. Further, this study planned to prepare the relative amine **(*S*)**-**5** isosteric structure, to gain information on the biological behavior of the methyl 9-hydroxystearate and methyl 9-aminostearate. This study decided to test also the biological activity of the azide **(*S*)**-**4** species, as precursor of the mentioned amine species.

**Scheme 1:**
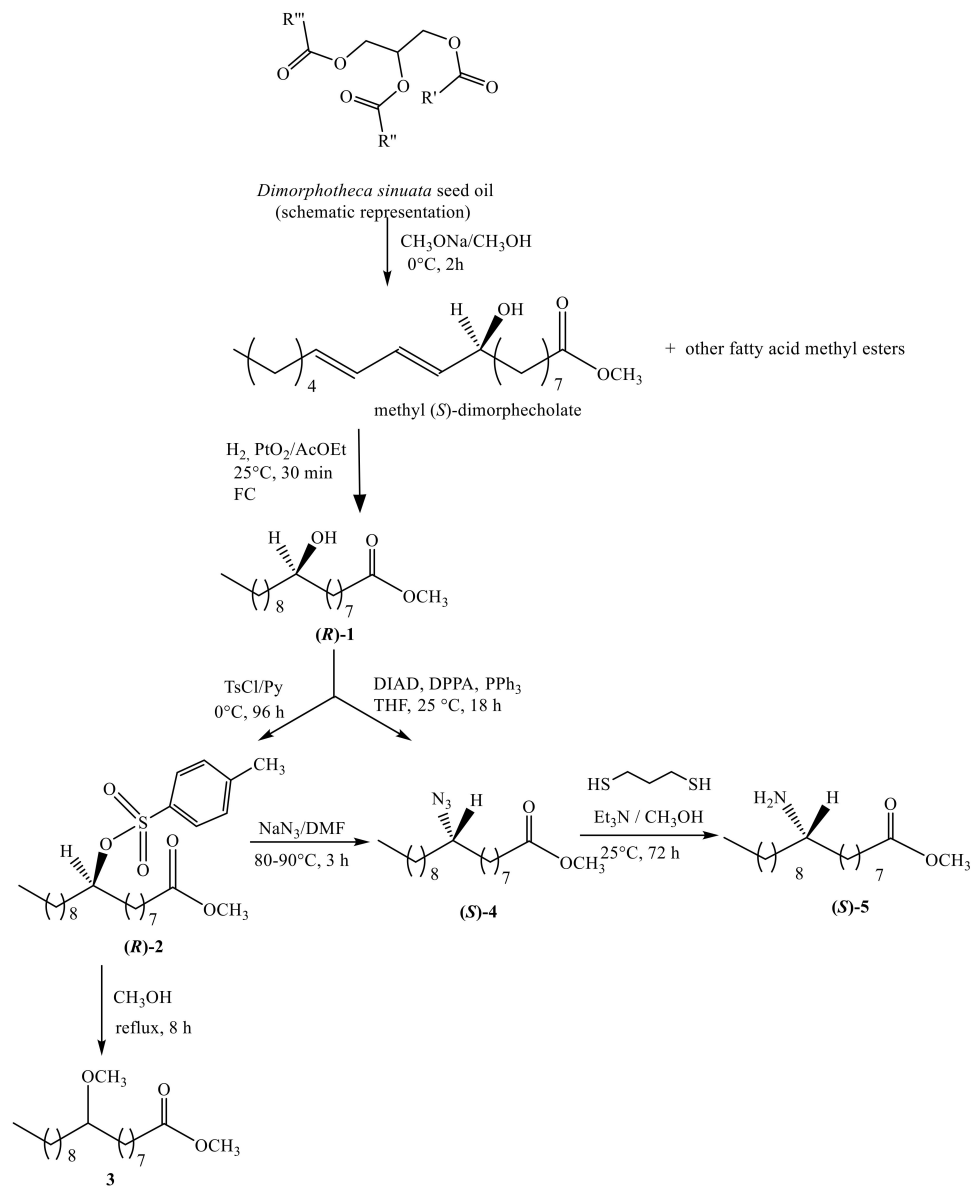
Synthetic routes to 9-HSA derivatives.

All compounds shown in Figure 2 were obtained starting from methyl (9*R*)-9-hydroxystearate [**(*R*)-1**], which in turn was obtained from *Dimorphotheca sinuata* seeds, which is oil, as well as the seeds of other plants of the genus Dimorphotheca. These contain high amounts of (9*S*,10*E*,12*E*)-9-hydroxyoctadeca-10,12-dienoic acid [(*S*)-dimorphecholic acid] [11], a precursor of **(*R*)**-**1** and of **(*R*)**-9-HSA. The natural availability of (*S*)-dimorphecholic acid is a green and efficient way to obtain 9-HSA derivatives with the C-9 chiral carbon atom in enantiopure form, permitting all the difficulties exhibited by alternative synthetic methods [12] to be overcome. Scheme 1 shows the synthetic strategy adopted to prepare compounds **(*R*)-1**, **(R)**-**2**, **3**, **(*S*)**-**4** and **(*S*)**-**5**.

The procedure appears to be the first reaction of transmethylation of the triglycerides, obtained from the extraction of the seeds and subsequent hydrogenation over the Adam’s catalyst. After purification of the crude product by silica gel chromatography, the (*9R*)-methyl-9-hydroxystearate [**(*R*)**-**1**] was isolated and its optical purity was ascertained by ^1^H NMR upon derivatization with (*R*)*-O-*acetylmandelic acid [10].

Compound **(*R*)**-**1** was then transformed by treatment with tosyl chloride into **(*R*)**-**2**. The reaction of **(*R*)**-**2** with refluxing methanol produced the methyl ether **3**. For the latter, the absolute configuration of the C-9 chiral center is not defined due to the possibility of occurrence, together with an inversion of the configuration, and also of a S_N_1 mechanism producing racemization. The addition to **(*R*)**-**2** of sodium azide in *N*,*N*-dimethylformamide (DMF) at 80–90 °C produced the azide **(*S*)**-**4**. However, due to difficulties of the work-up and purification steps, an alternative method was adopted by reacting **(*R*)**-**2** with diphenylphosphoryl azide (DPPA) under Mitsunobu conditions yielding **(*S*)**-**4** in satisfactory yield (77%).

**Scheme 2:**
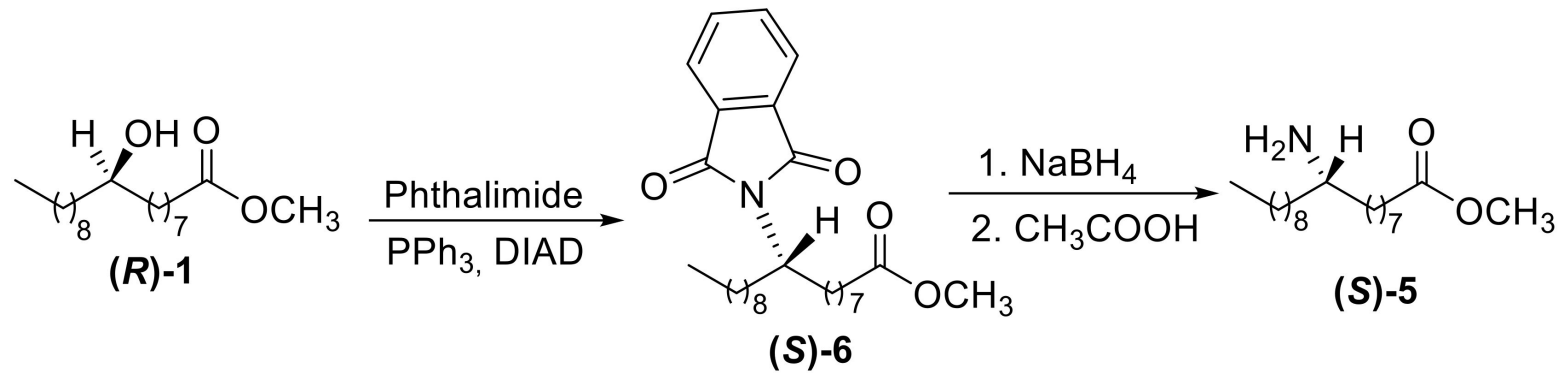
Synthetic pathway to obtain aminoderivative **(*S*)-5** from **(*R*)-1**.

The last step was devoted to the reduction of the azide. The adoption of Bayley’s method (1,3-propandithiol and Et_3_N) [13] suffered a particularly hard work-up: After the separation of the amine from by-products through immobilization on the Dowex resin and subsequent release with triethylamine, a further treatment by liquid-liquid extraction was required and the desired amine was recovered in 13% yield. This encouraged the study to try the alternative synthetic route shown in Scheme 2 that gave, after purification, the amine **(*S*)**-**5** in 36% overall yield from **(*R*)**-**1**.

**Scheme 3:**
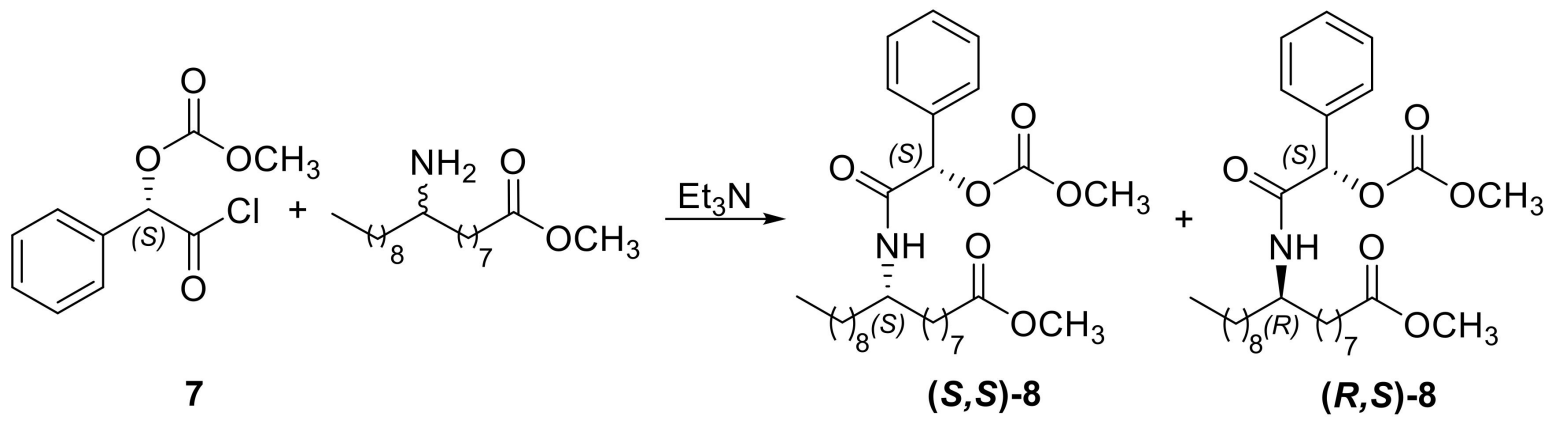
Derivatization of the amine **5** with (*S*)-2-chloro-2-oxo-1-phenylethyl methyl carbonate (**7**) to determine the **(*S,S*)**-**8**/**(*R,S*)**-**8** diastereomeric ratio.

The enantiopurity of the new amine **(*S*)**-**5** was ascertained by reacting it with the acyl chloride of the (*S*)*-O-*acetylmandelic acid (**7**) (Scheme 3) and by calculating, from the ^1^H-NMR spectrum of the crude, the ratio between the two diastereomeric amides **(*S,S*)-8** and **(*R,S*)**-**8**. The signals were falling at 3.669 and 3.665 ppm, belonging to the **(*S,S*)** and (***R,S***) diastereomer, respectively, from which a 9/1 *dr* was calculated, which was an indication of an almost complete inversion of the configuration during synthesis (see Figures S1–S17). This was confirmed by adding a little amount of the **(*R,S*)**-**8** diastereomer (obtained as described below) to the above mixture: A sensitive increase of the area of the peak at 3.665 ppm was observed.

As reported in Scheme 1, several steps of the synthetic pathway imply the inversion of configuration on C-9 producing the compounds **4** and **5** with the (*S*) configuration. Since the two enantiomers showed distinct antiproliferative activity by the preliminary tests in the case of 9-HSA, this study planned to prepare the opposite enantiomers of those compounds that, in preliminary tests, were biologically active. Thus, **(*S*)**-**1** and **(*R*)**-**5** were prepared in order to compare the biological activity of the two enantiomers (Figure 2), and to evaluate the trend in biological activity ascribed to both the isosters with same configuration.

**Scheme 4:**
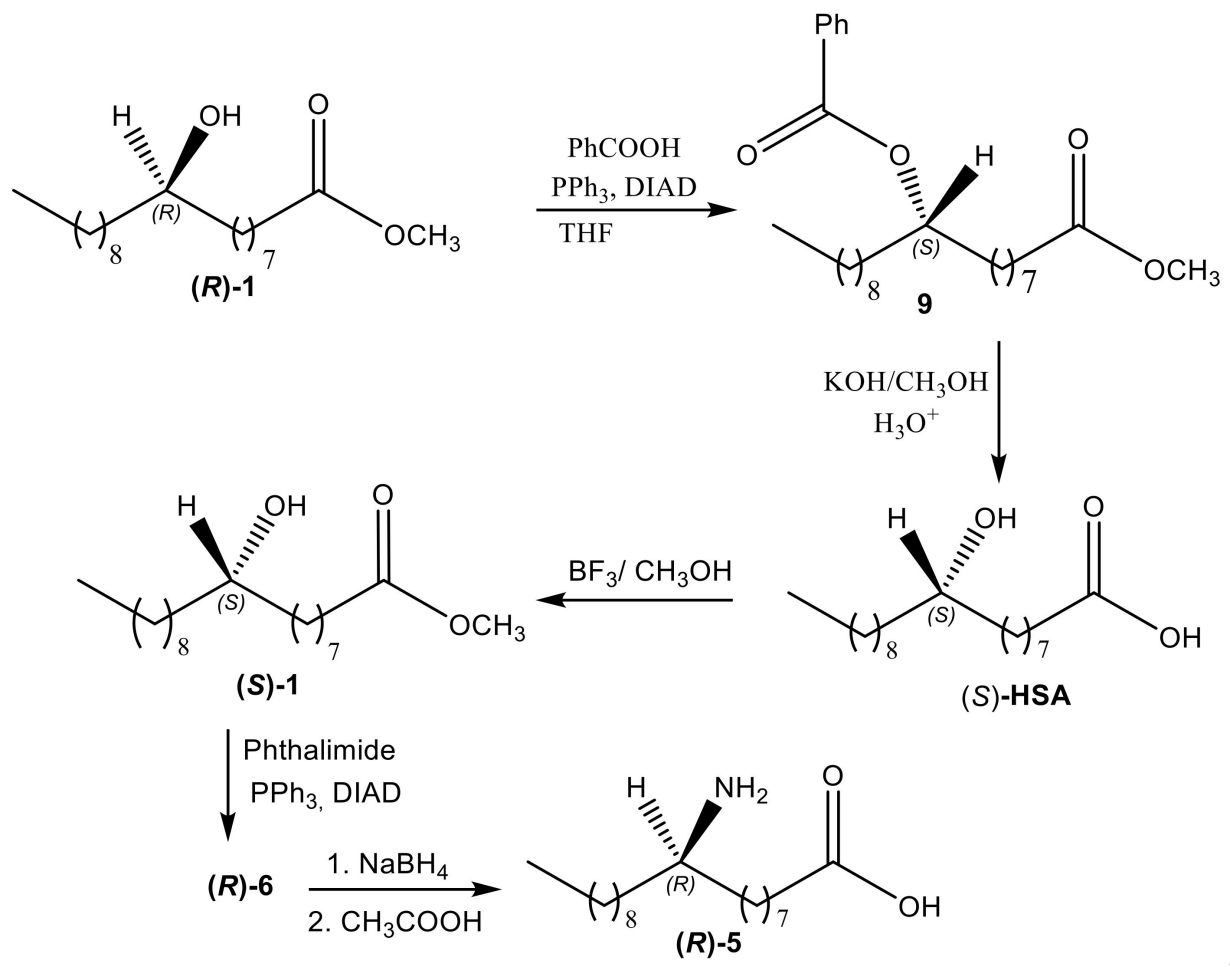
Synthesis of (*S*)-1 and (*R*)-5.

The procedure utilized is shown in Scheme 4: **(*S*)**-**1** was obtained by the inversion of the chiral center of **(*R*)**-**1** through the Mitsunobu reaction followed by the treatment of the intermediate **9** with methanolic KOH and esterification with BF_3_/CH_3_OH. Then, **(*R*)**-**5** was obtained from **(*S*)**-**1** by the same procedure depicted in Scheme 2 to obtain the opposite enantiomer.

The enantiopurity degree of **(*S*)**-**1** was measured [11,14] by reacting it with (*R*)-(−)*-O-*acetylmandelic acid: A diastereomeric ratio > 5/95 between the (*R,R*) and (*S,R*) diastereomers was calculated from the ^1^H-NMR of the crude reaction mixture. Analogously, the **(*R*)**-**5** enantiopurity degree was evaluated as a 10/90 diastereomeric ratio, by analyzing ^1^H-NMR spectrum of the reaction with the acyl chloride of (*S*)-*O*-acetylmandelic acid.

### 2.2. Biological Activity

#### 2.2.1. Preliminary Biological Activity of (*R*)-**2**, 3, (*S*)-**4**, and (*S*)-**5**

In order to gain information on the effect of the change of the group bound to C-9, preliminary biological tests on IC_50_ value for compounds **(*R*)**-**1**, **(*R*)**-**2**, **3**, **(*S*)**-**4**, and **(*S*)**-**5** were carried out. From the MTT tests, the only active compounds were the methyl ester **(*R*)**-**1** and the amino derivative **(*S*)**-**5** (see below). The absence of significant activity in the case of compounds functionalized on C-9 with groups not able to make hydrogen bonds might be considered as a strong indication of the importance of the presence of a group bearing hydrogen atoms bound to a heteroatom on this position. The results obtained with **(*R*)**-**1** and **(*S*)**-**5** encouraged this study to also synthesize the corresponding enantiomer in order to compare their biological effects.

#### 2.2.2. Effect of Methyl (9*R*)-9-hydroxystearate [(*R*)-1], Methyl (9*S*)-9-hydroxystearate [(*S*)-1], Methyl (9*R*)-9-aminostearate [(*R*)-5] and Methyl (9*S*)-9-aminostearate [(*S*)-5] on Proliferation of HT29

**Figure 3:**
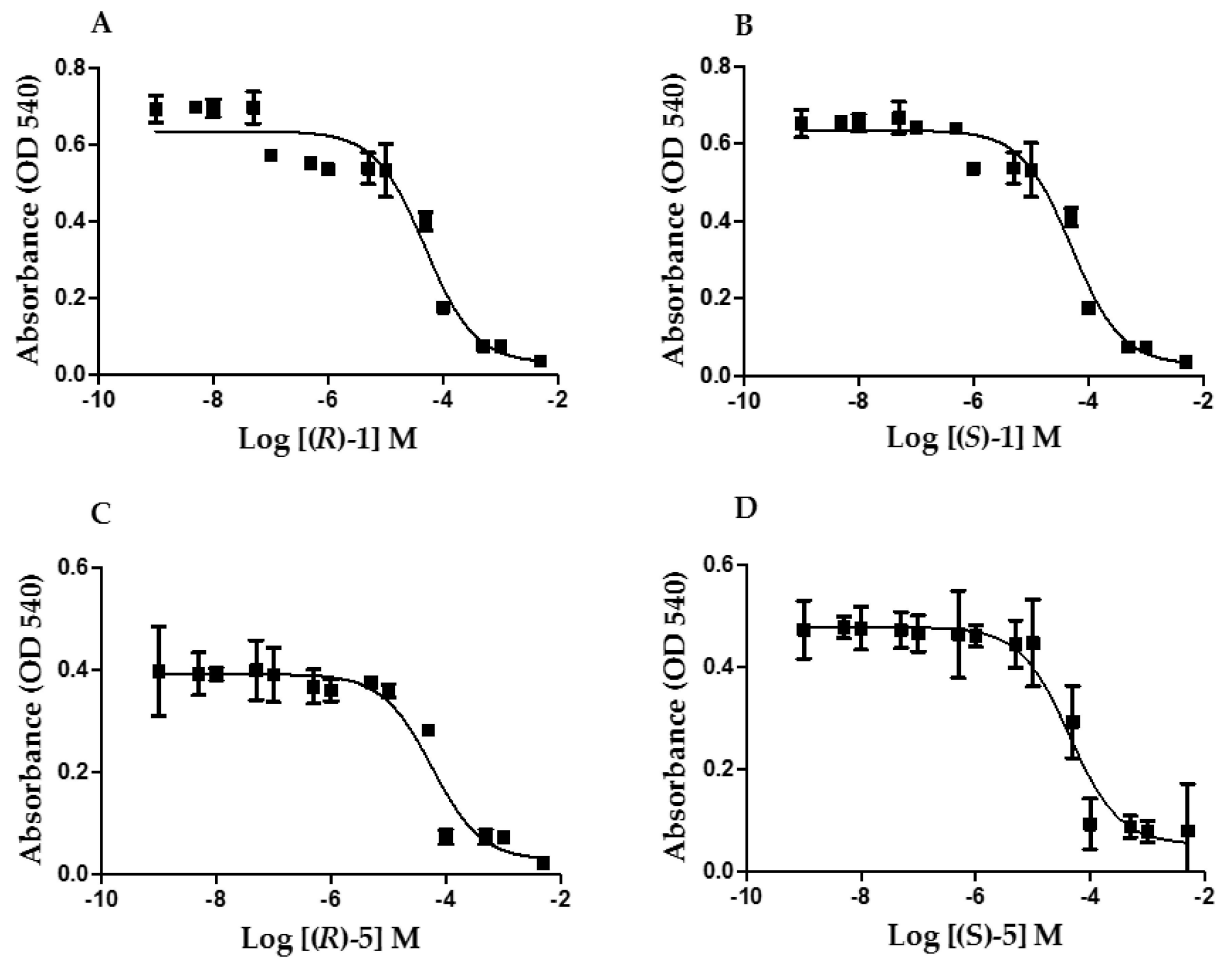
Dose response curves of HT29 cell viability upon treatment with different concentrations of (**A**) **(*R*)**-**1**, of (**B**) **(*S*)**-**1**, of (**C**) **(*R*)**-**5** and (**D**) **(*S*)**-**5**.

The in vitro IC_50_ growth inhibitory concentration was determined for **(*R*)**-**1**, **(*S*)**-**1**, **(*R*)-5** and **(*S*)**-**5**, incubating the HT29 with increasing concentrations of the compounds for 24 h. The data obtained from the MTT analyses were examined to assess the concentration of compounds required for 50% inhibition of cell viability (IC_50_): The values corresponded to 49 ± 1.3 *µ*M for (***R*)**-**1,** 51 ± 1.1 *µ*M for **(*S*)-1,** 57 ± 2.1 *µ*M for **(*R*)-5** and 43 ± 4.5 µM for **(*S*)**-**5** (Figure 3).

**Figure 4:**
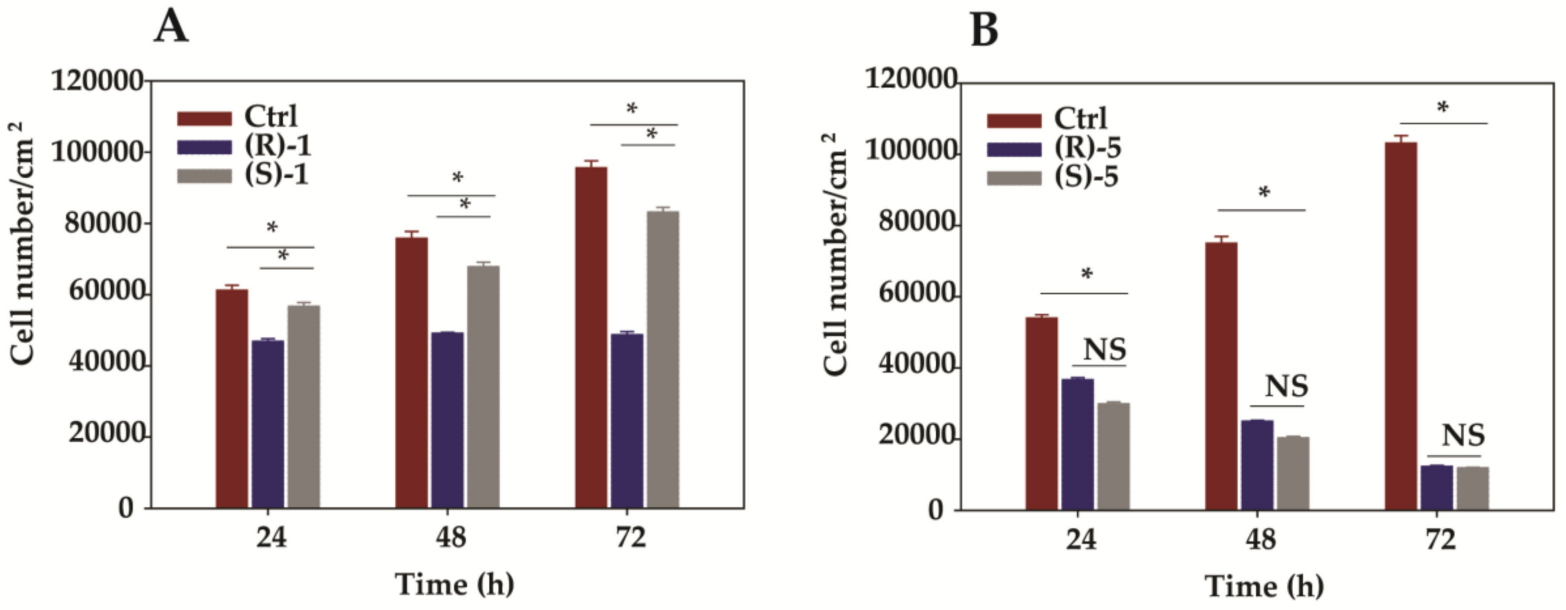
Effects on cell proliferation. (**A**) Antiproliferative effect of **(*R*)**-**1** and **(*S*)**-**1** on HT29 at 24, 48, and 72 h of treatment as compared with the control. (**B**) Antiproliferative effect of **(*R*)**-**5** or **(*S*)**-**5** in the same experimental conditions. The analysis was carried out by two-way analysis of variance (ANOVA) followed by the Bonferroni multiple comparisons. The differences of at least *p* < 0.05 were considered significant. Statistical analysis was carried out using Prism GraphPad software.

The effects on HT29 cell proliferation after treatment with (***R*)**-**1** and **(*S*)**-**1** and with (*R*)-5 and (*S*)-5 are reported in Figure 4. In panel A, the effects of 49 µM (*R*)-1 and 51 µM (*S*)-1 are reported. The results for the **(*R*)**-**1** and **(*S*)**-1 treatment show a significantly greater effect for the (*R*)-enantiomer, recalling the already reported behavior of the two enantiomers of 9-hydroxystearic acid. In panel B, the effects for (*R*)-5 and (*S*)-5 at the respective concentrations of 57 µM and 43 µM are reported: In this case, after 24 h of treatment, the cell proliferation strongly reduced, and after 48 h and 72 h of treatment, the cell numbers still diminished, indicating a continuous cytotoxic effect.

These findings prompted this study to gain more information on the biological effect of the methyl (9*R*)-9-hydroxystearate **(*R*)**-**1**.

#### 2.2.3. Cell Cycle Analysis

**Figure 5:**
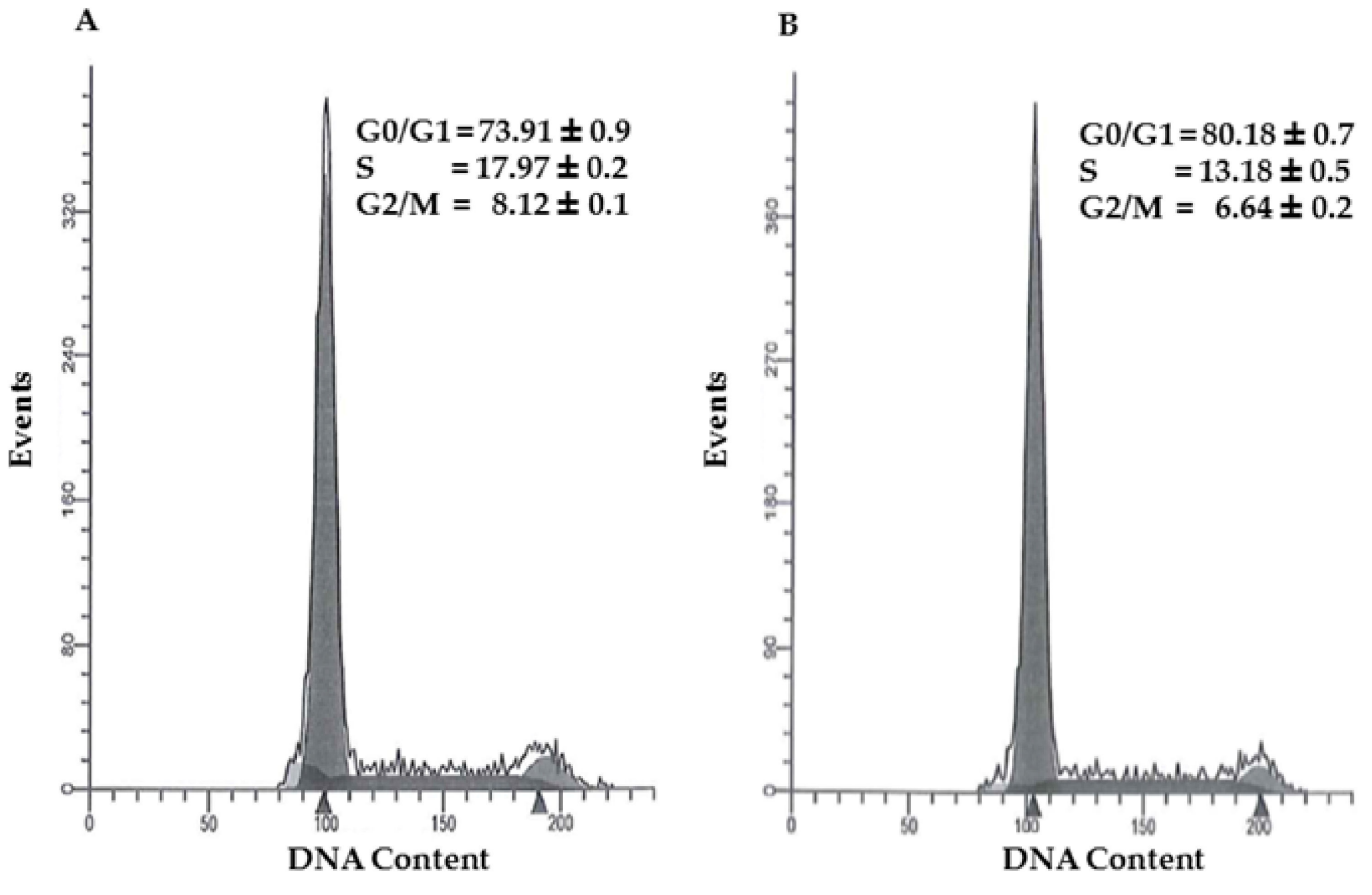
Effect of **(*R*)**-**1** on cell cycle. The cells were incubated for 48 h with the vehicle (**A**) or with 49 µM of **(*R*)-1** (**B**), afterward the cell cycle distribution was determined by flow cytometry. The two panels report the cytofluorimeter outputs obtained in one typical experiment repeated twice with similar results.

In order to assess whether the antiproliferative effect of (*R*)-1 was associated with the interference of the cell cycle progression, DNA profiles of cultured cells were examined by flow cytometry. The cells were exposed to the compound for 48 h prior to processing and analysis. As shown in Figure 5, the exposure to (***R*)**-**1** resulted in an increase in the number of the G0/G1 (80.18 ± 0.7% versus 73.91 ± 0.9%) phase, while reducing their number in the S (13.18 ± 0.5% versus 17.97 ± 0.2%) and G2/M (6.64 ± 0.2%versus 8.12 ± 0.1%) phases, thus indicating that the treatment leads to a cell cycle arrest. The flow cytometric data correlates with cell counting over time. Indeed, the administration of **(R)**-**1** reduced the number of cells by 23% at 24 h, by 35% at 48 h and by 49% at 72 from the treatment, indicating that the cells did not duplicate anymore.

#### 2.2.4. Effect of (*R*)-1 on Histone Acetylation

Since the above findings parallel those found for the parent (*R*)-9-HSA [11], an investigation of whether the molecular target might be histone deacetylase, as in the case of 9-HSA, was undertaken.

**Figure 6:**
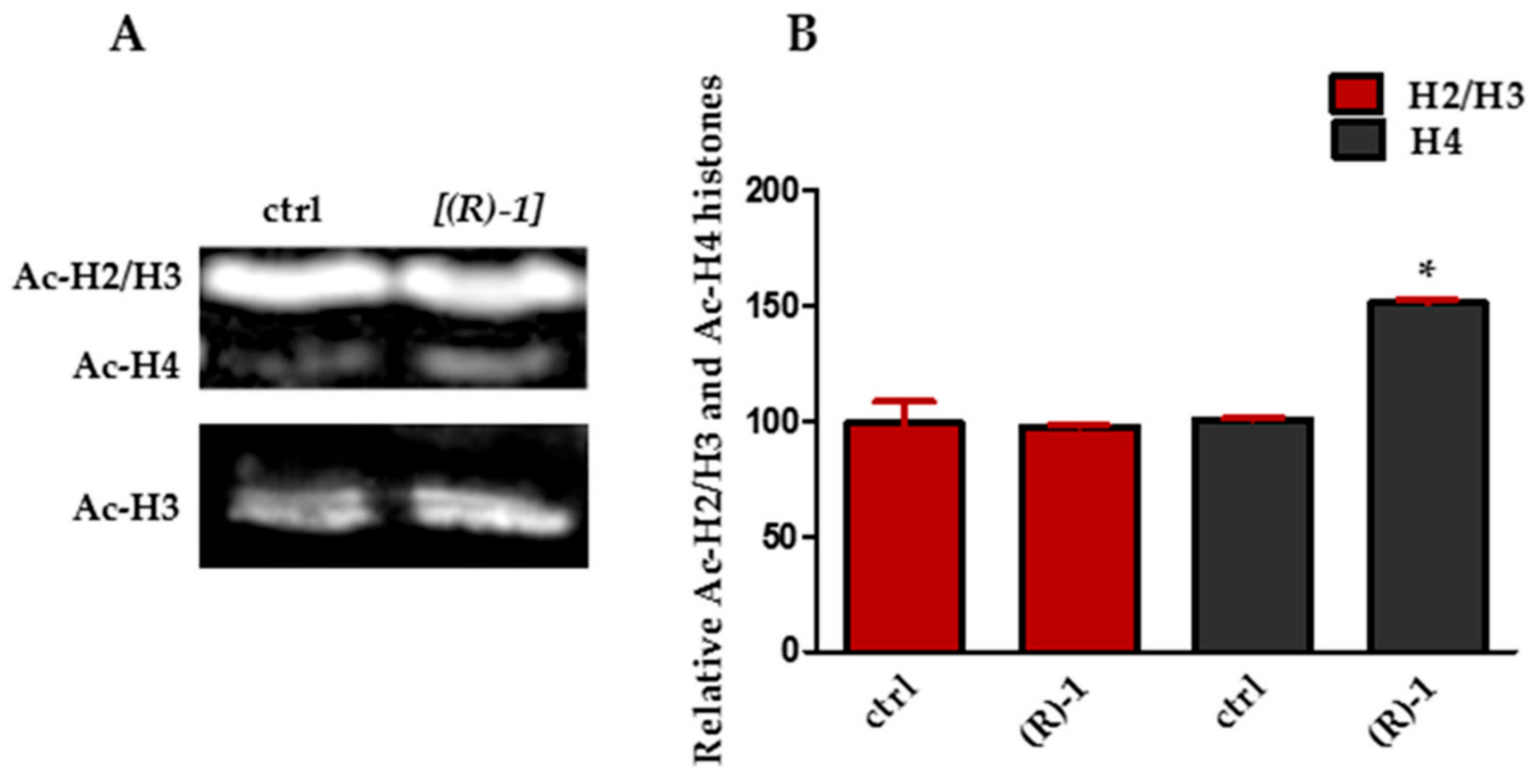
Effect of **(*R*)**-**1** on histone H4 acetylation. (**A**) The cells were cultured for 6 h in the absence (control) or presence of 49 µM (***R*)**-**1**. The cell nuclear extracts were prepared and subjected to a western blot analysis for acetylated (ac) histone H2/H3, histone H4 and histone H3 as loading controls. (**B**) A representative experiment, repeated three times with similar results and densitometric analysis of the bands (mean ± SEM; *n* = 3), are shown. * *p* < 0.05.

To identify the state of histones acetylation, these proteins were extracted from the control HT29 and treated for 6 h with 49 µM of **(*R*)**-**1**. Acetylation was detected by a western blot using an anti-acetyl lysine monoclonal antibody. The histone acetylation signals were quantified by densitometry and normalized on histone H3. As shown in Figure 6, the treatment with **(*R*)**-**1** for 6 h increased histone H4 acetylation by 50%, while the acetylation status of histones H2/H3 did not change.

2.2.5. (*R*)-1 Induces Gene and Relative Protein Expression Modulations

**Figure 7:**
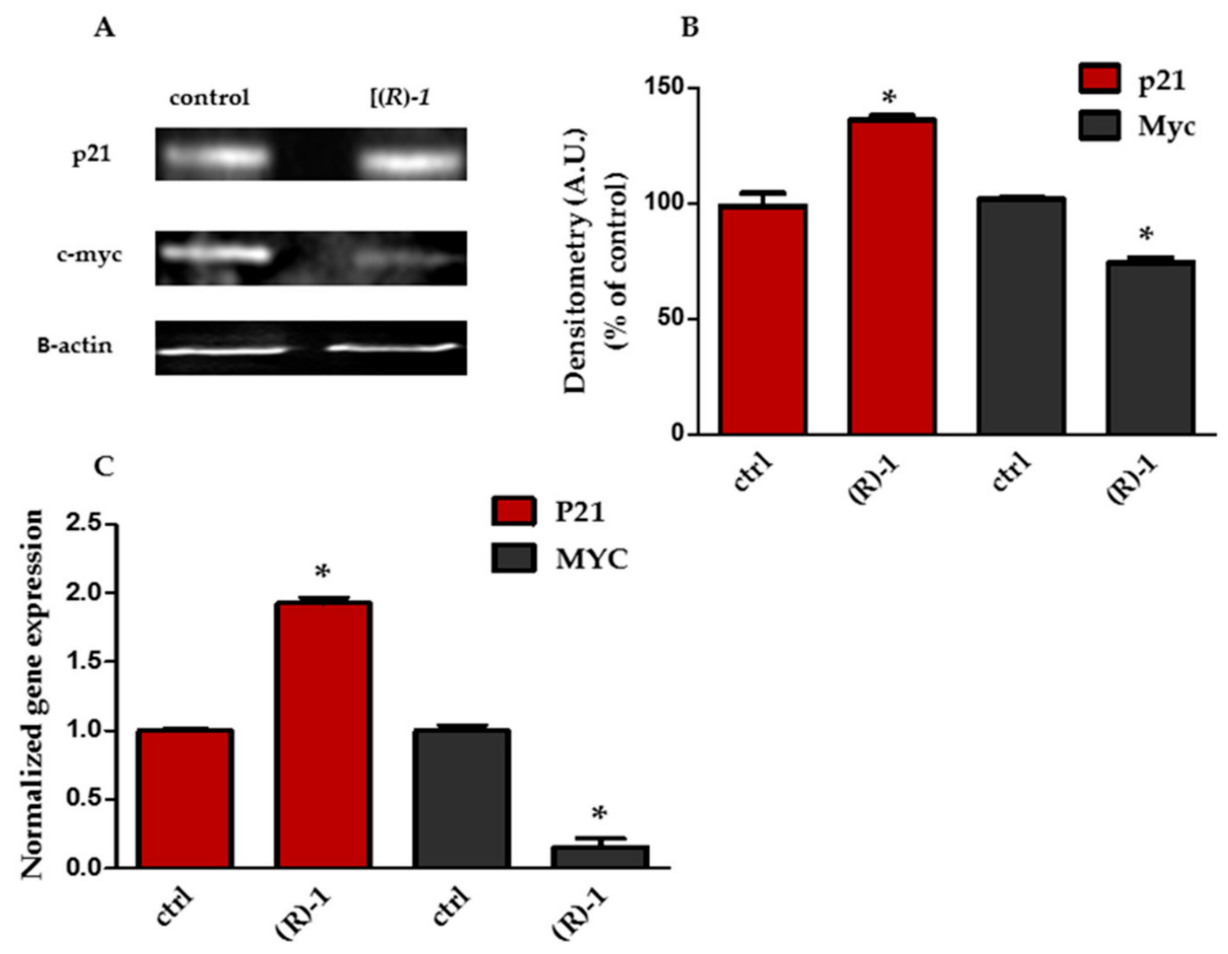
The effects of **(*R*)**-**1** on the expression of cell growth regulatory genes and proteins in HT29 colon cancer cell line. **A**) Representative western blots for p21 and MYC in HT29 cells following 6 h treatment with **(*R*)**-**1**. β-actin was used as the control. **B**) Densitometry from western blots for HT29 following 6 h treatment with **(*R*)-1**. Protein quantification was normalized to the β-actin band. The mean ± SEM, *n* = 3, and * *p* < 0.05. **C**) Changes in mRNA expression of P21 and MYC. GAPDH were used as a control gene. CT values were obtained, the data was normalized against GAPDH and the fold change was calculated by ∆CT method. The data are the mean ± SEM of three independent experiments. * *p* < 0.05.

The induction of p21 is one of the common phenomena observed after treatment with HDAC inhibitors such as TSA, NaBu, SAHA or 9-HSA [7,15,16,17]. Myc is a transcription factor whose activity is causally involved in cancers, principally through its capacity to drive tumor cell proliferation and promote angiogenesis, invasion, and metastasis [18,19,20,21]. The downregulation of Myc is an essential part of the anti-proliferative response to differentiation signals. Thus, a western blot analysis was performed to assess the expression of p21 and Myc in HT29 cells after **(*R*)**-**1** treatment. As expected, p21 was induced by the treatment with **(*R*)**-**1** compared to its expression level in the untreated control cells, while the Myc expression decreased significantly by **(*R*)**-**1** treatment (Figure 7A,B).

The observed cytostatic effects of **(*R*)**-**1** transcription levels of P21 and MYCBP (MYC) were analysed by quantitative Real Time-Polymerase Chain Reaction (RT-PCR. The genes were analysed after 6 h of treatment. The qRT-PCR was performed on cDNA of the control and **(*R*)**-**1**-treated cells, and the ΔΔC_T_ method was used with the GAPDH gene as the housekeeping gene. The relative transcription levels, expressed as a means of fold changes, are reported in Figure 7C. **(*R*)-1** administration transcription significantly increases P21 gene at 6 h of treatment, while MYC is significantly reduced. 

## 3. Materials and Methods

### 3.1. Chemical Syntheses

Dimorphotheca sinuata L. seeds were bought from Galassi Sementi Srl (Gambettola, FC, Italy). The reagents used, unless stated otherwise, were purchased from Sigma-Aldrich (Milan, Italy). For flash chromatography (FC), silica gel 0.037–0.063 mm (Merck KGaA, Darmstadt, Germany) was used as the stationary phase. Thin layer chromatography (TLC) was carried out on silica gel 60 (Fluka Analytical, Buchs, Switzerland) and the spots were revealed, depending on the compound to be analyzed, using UV light, and an aqueous solution of (NH_4_)_6_MoO_24_ (2.5%) and (NH_4_)_4_Ce(SO_4_)_4_ (4%) in 10% H_2_SO_4_, a solution of ninhydrin 0.013 M in *n*-butanol/acetic acid, or a basic solution of potassium permanganate. Anhydrous THF was freshy distilled over sodium benzophenone ketyl. Pyridine, trimethylamine and methanol were dried by standard methods. The hydrogenation was carried out on a Parr hydrogenator model 3911EKX at a hydrogen gas pressure of approximately 40 psi. The nuclear magnetic resonance spectra (^1^H-NMR ^13^C-NMR,) were recorded at 25 °C on Varian spectrometers Gemini 300, Mercury 400, or Inova 600 (Varian, Palo Alto, CA, USA). The signal multiplicities were established by DEPT-135 experiments. The chemical shifts were referenced to the solvent (CDCl_3_, δ = 7.27 and 77.0 ppm for ^1^H and ^13^C-NMR, respectively). The GC-MS analyses were carried out by gas chromatograph directly interfaced with a mass selective detector (injection temperature: 250 °C; oven temperature was programmed as follows: 60 °C for 2 min, increased up to 260 °C at the rate of 20°/min, followed by 260 °C for 20 min; the carrier gas was helium, used at a flow rate of 1 mL; the transfer line temperature was 280 °C; the ionization was obtained by electron impact (EI); the acquisition range was 50–500 *m*/*z*). The ESI-MS spectra were recorded using a Waters 2Q 4000 instrument (Waters Corporation, Milford, MA, USA). The melting points were measured on a Büchi apparatus (Stone, Staffs, UK) and were not corrected. The Fourier transform infrared (FT-IR) spectra of the organic compounds were recorded using a Perkin-Elmer FT-IR MOD 1600 spectrophotometer (Norwalk, CT, USA).

#### 3.1.1. Synthesis of Methyl (*9R*)-9-hydroxyoctadecanoate [**(*R*)-1**]

*Dimorphotheca sinuata* L. seeds (12.0 g) were ground and suspended in a CHCl_3_/MeOH mixture (2:1 *v*/*v*, 200 mL) and the mixture was kept under nitrogen atmosphere, in the dark, and stirred for 24 h. After filtration and washing of the solid with CHCl_3_ (3 × 20 mL), 20 mL of 0.1 M HCl and 0.1 M NaCl aqueous solution was added to the green solution. After extraction with CHCl_3_ (3 × 15 mL), the combined organic layers were dried over anhydrous MgSO_4_. After filtration and solvent removal in vacuo at 30 °C, 4.0 g of residue was recovered. The crude yellow oil was dissolved in CH_3_OH (40 mL) under nitrogen atmosphere and the solution was kept at 0 °C by immersion in an external water/ice bath. Then, CH_3_ONa (2.16 g) was added and the mixture was magnetically stirred at 0 °C for 2 h. The mixture was acidified to pH 4.0 and extracted with *n*-hexane (3 × 20 mL). The combined organic layers were dried over anhydrous MgSO_4_. After filtration, the solvent was removed under reduced pressure and the residue (2.2 g) dissolved in 10 mL of ethyl acetate and transferred into the hydrogenation vessel. A catalytic amount of PtO_2_ (Adam’s catalyst) was added and the mixture was subjected to hydrogenation (40 psi H_2_ pressure). The reaction course was monitored by ^1^H-NMR spectroscopy. Usually, the hydrogenation is complete after approximately 40 min. The mixture was then filtered over celite and the solution concentrated under vacuum. Flash chromatography (eluent: petroleum ether/diethyl ether 7/3) of the residue gave **methyl (*9R*)-9-hydroxyoctadecanoate** [**(*R*)-1**] (C_19_H_38_O_3_) as a white solid (0.74 g), m.p.: 50–51 °C (Lit. [22]: 50–51.5 °C). ^1^H-NMR (600 MHz, CDCl_3_): δ (ppm) = 3.67 (s, 3 H, OCH_3_), 3.61–3.55 (m, 1 H, CHOH), 2.30 (t, 2 H, *J* = 7.6 Hz, CH_2_COO), 1.62 (quint, 2 H, *J* = 7.4 Hz, CH_2_CH_2_COO), 1.48–1.21 (m, 27 H, incl. OH), 0.88 (t, 3 H, *J* = 7.0 Hz, CH_3_). ^13^C-NMR (100.6 MHz, CDCl_3_) δ (ppm) = 174.1, 71.7, 51.3, 37.4, 37.3, 33.9, 31.8, 29.6, 29.56, 29.5, 29.4, 29.2, 29.1, 28.9, 25.5, 25.4, 24.8, 22.6, 14.05. IR (CHCl_3_): 3427, 1728 cm^−1^. MS (EI) *m*/*z* (%): 283 (M^+^ − OCH_3_, 2), 264 (4), 187(45), 159 (11), 158 (53), 155 (100), 129 (6), 115 (18), 109 (14), 87 (50), 74 (33), 69 (18), 55 (31). The enantiomeric excess was determined by derivatization with (*R*)-(–)*-O-*acetyl mandelic acid as previously reported [12,14] and was found to be 80%.

#### 3.1.2. Synthesis of Methyl (*R*)-9-(tosyloxy)octadecanoate [**(*R*)-2**]

Methyl (*9R*)-9-Hydroxyoctadecanoate [**(*R*)-1**, 0.190 g (0.60 mmol], 0.607 g (3.18 mmol) of TsCl and 10 mL of anhydrous pyridine were kept at 0 °C for 1 h then at 4 °C. The reaction was monitored by TLC (*n*-hexane/AcOEt 1:1, R_F_ = 0.54) and, when it was complete (after approximately 5 days), it was quenched with water (40 mL). The mixture was extracted with Et_2_O and the combined organic layers were acidified with HCl 1 M. The organic layer was dried over anhydrous MgSO_4_, filtered and concentrated under reduced pressure. Compound **(*R*)**-**2** (C_26_H_44_O_5S_) was obtained as colorless oil in 70% yield. ^1^H-NMR (300 MHz, CDCl_3_): δ (ppm) = 0.87 (t, *J* = 6.9 Hz, 3 H, CH_3_), 1.13–1.30 (m, 27 H, (CH_2_)_13_), 1.55 (m, 2 H, CH_2_CH_2_COOCH_3_), 2.27 (t, *J* = 7.56 Hz, 2 H, CH_2_COOCH_3_), 2.42 (s, 3 H, PhCH_3_), 3.65 (s, 3 H, COOCH_3_), 4.52 (q, *J* = 6.1 Hz, 1 H CHOSO_2_), 7.31(d, 2 H, *J* = 4 Hz, phenyl), 7.77 (d, *J* = 4.4 Hz, 2 H, phenyl). ^13^C-NMR (75.44 MHz, CDCl_3_): δ (ppm) = 174.3, 144.3, 134.8, 129.6, 127.7, 84.5, 51.4, 34.1 (two signals overlapped), 34.0, 31.9, 29.43, 29.38, 29.3(two signals overlapped), 29.1, 29.0, 28.9, 24.8, 24.7, 24.6, 22.6, 21.6, 14.1. ESI-MS (*m*/*z*): 481 [M + Na]^+^, 469 [M + H]^+^

#### 3.1.3. Synthesis of Methyl 9-MethoxyOctadecanoate (**3**)

Compound **(*R*)**-**2** (0.167 g, 0.357 mmol) was dissolved in CH_3_OH (5 mL) and the solution was refluxed for 7 h. After removal of the solvent under reduced pressure, brine (20 mL) was added and the mixture was extracted with Et_2_O (4 × 20 mL). The combined organic layers were dried over anhydrous MgSO_4_. After filtration and concentration, the residue was purified by FC (eluent: *n*-hexane/CH_2_Cl_2_ from 6:4 to 1:1 to 3:7 to 2:8 until CH_2_Cl_2_). Pure methyl 9-methoxyoctadecanoate (**3**, (C_20_H_40_O_3_) was obtained in 55% (0.064 g, 0.195 mmol). ^1^H-NMR (300 MHz, CDCl_3_): δ (ppm) = 0.86 (t, *J* = 6.9 Hz, 3 H, CH_3_), 1.24–1.32 (m, 27 H, (CH_2_)_13_), 1.60 (m, 2 H, CH_2_CH_2_COOCH_3_), 2.29 (t, *J* = 7.49 Hz, 2 H, CH_2_COOCH*3*), 3.10 (m, 1 H, CHOCH_3_), 3.30 (s, 3 H, CHOCH_3_), 3.65 (s, 3 H, COOCH_3_). ^13^C-NMR (150.80 MHz, CDCl_3_): δ (ppm) = 174.3, 80.9, 56.3, 51.4, 34.1, 33.42, 33.41, 31.9, 29.9, 29.66, 29.65, 29.58, 29.3, 29.2, 29.1, 25.3, 25.26, 24.9, 22.7, 14.1. MS (EI) *m*/*z* (%): 327 (M^+^ − 1, 0.5) 297 (3), 264 (14), 222 (6), 201 (100), 171 (50), 137 (29), 123 (8), 109 (19), 97 (43), 83 (72), 69 (57), 55 (83).

#### 3.1.4. Synthesis of Methyl (9*S*)-9-azidooctadecanoate [**(*S*)-4**]

Method 1:

Compounds (***R*)-2** (0.147 g, 0.314 mmol), NaN_3_ (0.102 g, 1.57 mmol), and DMF (10 mL) were heated at reflux for 3 h. The mixture was treated once with brine (20 mL) then extracted with Et_2_O (3 × 15 mL), then two folds with water (30 mL and 50 mL) and each time extracted with Et_2_O (3 × 15 mL), finally with brine (20 mL) then extracted with Et_2_O (3 × 15 mL). Each time the organic layer was dried over anhydrous MgSO_4_ and filtered: The absence of DMF was checked. The combined organic layers were concentrated and the residue chromatographed on silica gel (eluent: 7:3 *n*-hexane/AcOEt). Compound **(*S*)-4** (C_19_H_37_N_3_O_2_, 0.064 g, 58%) was recovered as colorless oil. ^1^H-NMR (300 MHz, CDCl_3_): δ (ppm) = 0.88 (t, *J* = 6.6 Hz, 3 H, CH_3_), 1.24–1.33 (m, 26 H, (CH_2_)_13_), 1.61 (m, 2 H, CH_2_CH_2_COOCH_3_), 2.30 (t, *J* = 7.9 Hz, 2 H, CH_2_COOCH_3_), 3.21(q, *J* = 6.1 Hz, 1 H, CHN_3_), 3.65 (s, 3 H, COOCH_3_). ^13^C-NMR (150.80 MHz, CDCl_3_): δ (ppm) =174.3, 63.1, 51.4, 34.4, 34.3, 34.0, 31.9, 30.3, 29.5, 29.4, 29.3, 29.2, 29.1, 29.0, 26.1, 26.0, 24.9, 22.6, 14.1; IR-Neat (cm^−1^): 2928.0, 2856.0, 2361.2, 2098.7, 1742.6, 1465.4, 1257.5, 1171.1; ESI-MS (*m*/*z*): 352 [M + Na]^+^, 340 [M + H]^+^

Method 2:

Compound **(*R*)-1** (0.230 g, 0.732 mmol) was dissolved in anhydrous THF (7.5 mL) and under nitrogen atmosphere. PPh_3_ (0.398 g, 1.52 mmol) and DIAD 94% (300 µL, 1.52 mmol) were added and the yellow mixture was stirred. After a few minutes, DPPA 97% (330 µL, 1.52 mmol) was added. The solution became cloudy and after 18 h, had come clear. The crude was subjected to FC (eluent: *n*-hexane/AcOEt 9:1) and pure **(*S*)-4** (0.190 g, 77%) was recovered.

#### 3.1.5. Synthesis of Methyl (9*S*)-9-aminooctadecanoate [**(*S*)-5**]

Compound **(*R*)-1** (0.222 g, 0.70 mmol) was dissolved in anhydrous THF (8.0 mL) in a flame dried apparatus immersed in an ice-bath and kept under nitrogen atmosphere. PPh_3_ (0.103 g (0,70 mmol) and phthalimide (0.103 g (0,70 mmol) were added. Through a funnel, DIAD (0.2 mL, 0.70 mmol) in 2.0 mL of THF was added dropwise. After 12 h, the reaction appeared complete (TLC: petroleum light/diethyl ether 6:4). The reaction mixture was concentrated. FC on silica gel (light petroleum/diethyl ether: 20:1) of the residue gave 0.280 g (0.63 mmol, 90% yield) of the intermediate (9*S*)-9-(1,3-dioxo-1,3-dihydro-2H-isoindol-2-yl) octadecanoate [**(*S*)**-**6**] (C_27_H_41_NO_4_) as a colorless oil:

^1^H-NMR (400 MHz, CDCl_3_), δ (ppm) = 7.83–7.79 (m, 2 H), 7.71–7.68 (m, 2 H), 4.26–4.06 (m, 1 H), 3.63 (s, 3 H, OCH_3_), 2.25 (t, *J* = 7.43 Hz, 2 H), 2.11–1.97 (m, 2 H), 1.74–1.61 (m, 2 H), 1.61–1.49 (m, 2 H), 1.32–1.14 (m, 22 H), 0.84 (t, *J* = 6.97 Hz, 3 H, CH_3_). ^13^C-NMR (100.56 MHz, CDCl_3_), δ (ppm) = 174.2, 168.8, 133.8, 131.8, 123.0, 52.2, 51.4, 34.0, 32.5, 32.4, 31.8, 29.45, 29.43, 29.23, 29.21, 29.04 (2 signals overlapped), 29.0, 26.6, 26.5, 24.8, 22.6, 14.1. ESI-MS (*m*/*z*): 444 [M + H]^+^.

Sodium borohydride (0.06 g, 1.6 mmol) in 2-propanol (3.0 mL) was added to a solution of the compound **(*S*)-6** (0.131 g, 0.29 mmol) and the mixture was magnetically stirred at room temperature for 12 h. Glacial acetic acid (0.3 mL) was added and the mixture was heated at 80 °C for 2 h. After concentration, water (5 mL), and then saturated aqueous solution of NaHCO_3_ (5 mL) were added. After extraction with ethyl acetate (3 × 10 mL), the combined organic layer was dried over anhydrous magnesium sulfate. After filtration and removal of the solvent under reduced pressure, the crude was subjected to FC (eluent: CH_2_Cl_2_/CH_3_OH 10/1), the spots being evidenced with ninhydrin stain. Compound **(*S*)-5** (0.027 g, 0.09 mmol) was obtained in a 30% yield. An alternative method was as follows: Hydrazine hydrate (0.16 mL, 3.2 mmol) was added to a solution of compound **(*S*)-6** (0.280 g, 0.63 mmol) in ethanol (5 mL) and the mixture was heated and refluxed for 3 h. The formation of a white solid was observed. After cooling, 1 M HCl was added until pH = 6. Then, the solution was filtered on a Buchner funnel. The mixture was treated with saturated solution of NaHCO_3_ until reaching pH ~ 9 and then it was extracted with CH_2_Cl_2_ (4 × 10 mL) and dried over anhydrous MgSO_4_. After filtration and removal of the solvent under reduced pressure, the light yellow oil was subjected to FC (eluent: CH_2_Cl_2_/CH_3_OH 10/1), obtaining the product in 28% yield. Compound **(*S*)-5** (C_19_H_39_NO_2_): ^1^H-NMR (400 MHz, CDCl_3_): δ (ppm) = 0.88 (t, *J* = 6.9 Hz, 3 H, CH_3_), 1.24–1.32 (m, 26 H, (CH_2_)_13_), 1.58–1.65 (m, 2 H, CH_2_CH_2_COOCH_3_), 2.29 (t, *J* = 7.7 Hz, 2 H, CH_2_COOCH_3_), 3.13 (m, 1 H, CHNH_2_), 3.66 (s, 3 H, COOCH_3_), 8.30 (br.s, 2 H, NH_2_). ^13^C-NMR (100.56 MHz CDCl_3_): δ (ppm) = 174.2, 51.6, 51.4, 35.92, 35.86, 34.0, 31.9, 29.63, 29.55 (two signals overlapped), 29.4, 29.3, 29.12, 29.06, 25.8, 25.7, 24.9, 22.6, 14.1. MS (*m*/*z*): 282 (5), 267(1), 250(1), 207(3), 186(100), 156(99), 109(4), 97(2), 83(4), 70(5), 56(11). ESI-MS (*m*/*z*): 336 [M + Na]^+^, 314 [M + H]^+^; IR (cm^−1^): 3423.5, 2950.0, 2870.0, 1710, 1635.8.

The synthesis of **(*R*)-5** was carried out with the same procedure starting from **(*S*)-1**. 

#### 3.1.6. Synthesis of (*S*)-2-Chloro-2-oxo-1-phenylethyl Methyl Carbonate (**7**)

In a flame-dried apparatus and under nitrogen atmosphere, (*S*)*-O-*acetylmandelic acid (1.046 g, 5,4 mmol) and anhydrous CH_2_Cl_2_ (10 mL) were added. To the solution, oxalyl chloride (0.47 mL, 5.4 mmol) dissolved in anhydrous CH_2_Cl_2_ (2 mL) were added dropwise. After 2 h at room temperature and under magnetic stirring, the solvent was distilled at room pressure. The residue was distilled at 0.01 mmHg and the colorless oil with b.p.= 102–103 °C found to be compound **7** (C_10_H_9_ClO_4_).

^1^ H-NMR (300 MHz, CDCl_3_), δ (ppm): 7.59–7.37 (m, 5 H), 6.09 (s, 1 H), 2.22 (s, 3 H). ^13^ C-NMR (75.44 MHz, CDCl_3_), δ (ppm): 170.7, 169.8, 130.8, 130.2, 129.2, 128.4, 80.8, 20.4.

#### 3.1.7. Methyl (9*S*)-9-{[(2*S*)-2-(Acetyloxy)-2-Phenylacetyl]amino}octadecanoate [(*S*,*S*)-8] and Methyl (9*R*)-9-{[(2*S*)-2-(Acetyloxy)-2-Phenylacetyl]amino}octadecanoate [**(*R*,*S*)-8**]

The optical purity of **(*S*)-5** was determined by ^1^H-NMR analysis after derivatization with **7**, the acyl chloride of the (*S*)-(–)*-O-*acetylmandelic acid. Compound **7** (2.8 mg) in 0.5 mL of CD_2_Cl_2_ was put directly into the NMR spectroscopy tube and methyl 9(*S*)-9-aminostearate [**(S)-5**] (4 mg, 0.013 mmol) dissolved in CD_2_Cl_2_ (0.5 mL) was added. Then, triethylamine (2.7 µL) was added and the ^1^H-NMR spectrum of the crude was recorded. A diastereomeric ratio of ~9:1 between methyl (*9S*)-9-{[(*2S*)-2-(acetyloxy)-2-phenylacetyl]amino}octadecanoate and methyl (*9R*)-9-{[(*2S*)-2-(acetyloxy)-2-phenylacetyl]amino}octadecanoate was calculated from the integration of the signals belonging to the methoxy group of the two diastereomers (see spectrum SI-17). This relative ratio was calculated from the spectrum of the crude reaction mixture. The product ***(S,S)-*8** was then purified by preparative TLC on silica gel eluting with CH_2_Cl_2_ and scraping the spot with RF ~ 0.21. The pure compound ***(S,S)-*8** was extracted with CHCl_3_ and analyzed by NMR.

Methyl (9*S*)-9-{[(2*S*)-2-(acetyloxy)-2-phenylacetyl]amino}octadecanoate [(***S,S*)-8**] (C_29_H_47_NO_6_): ^1^H-NMR (600 MHz, CDCl_3_), δ (ppm): 7.45–7.41 (m, 2 H), 7.39–7.32 (m. 3H), 6.03 (s, 1 H), 5.66 (br. d., *J* = 9.44 Hz, 1 H, NH), 3.94–3.85 (m, 1 H. H-9), 3.669 (s, 3 H, OCH_3_), 2.29 (t, *J* = 7.61 Hz, 2 H), 2.19 (s, 3 H, CH_3_CO), 1.64–1.14 (m, 28 H), 0.88 (t, *J* = 7.04 Hz, 3 H, CH_3_).

The same procedure was used to determine the optical purity of methyl (9*R*)-9-{[(2*S*)-2-(acetyloxy)-2-phenylacetyl]amino}octadecanoate [**(*R,S*)**-**8**] (C_29_H_47_NO_6_): ^1^H-NMR (600 MHz, CDCl_3_), δ (ppm): 7.45–7.42 (m, 2 H), 7.39–7.33 (m. 3H), 6.03 (s, 1 H), 5.6 (br. d., *J* = 9.04 Hz, 1 H, NH), 3.95–3.85 (m, 1 H. H-9), 3.665 (s, 3 H, OCH_3_), 2.29 (t, *J* = 7.61 Hz, 2 H), 2.17 (s, 3 H, CH_3_CO), 1.64–1.14 (m, 28 H), 0.88 (t, *J* = 7.04 Hz, 3 H, CH_3_).

In order to verify if some signals of the two diastereisomers could be separated in the ^1^H-NMR spectrum, a mixture of (**S,S)**-**8** and **(*R,S*)**-**8** was prepared by mixing different amounts of the two species, previously purified by preparative TLC on silica gel (20 × 20 cm glass plates). The spectrum (600 MHz) showed separate methoxyl hydrogen signals (see Figures S1–S18).

Finally, to confirm the assignment of the signals at 3.669 and 3.665 ppm to the relative diastereomers, a small portion of purified **(*R,S*)**-**8** was added to a solution of a 9:1 diastereomeric mixture of **8**, and the peak at 3.665 ppm increased, as expected.

### 3.2. Cell Culture and Treatments

The human colon cancer cell line HT29 was obtained from American Type Culture Collection (Manassas, VA, USA). The cells were cultured in RPMI 1640 medium (Labtek Eurobio, Milan, Italy), supplemented with 10% FCS (Euroclone, Milan, Italy) and 2 mM l-glutamine (Sigma-Aldrich, Milan, Italy), at 37 °C and 5% CO_2_ atmosphere. These conditions were used in all cell incubation steps for the experiments described below. **(*R*)**-**1**, **(*S*)**-**1**, **(*R*)**-**5** and **(*S*)**-**5** were dissolved in ethanol to obtain a 33 mM stock solution then diluted in culture medium to obtain the required concentrations. The cells were plated at a density of 1.0 × 10^4^ cells/well in 6-well culture plates (Orange Scientific, Braine-l’Alleud, Belgium). Twenty-four hours after plating, **(*R*)**-**1** or **(*S*)**-**1** were added to a final concentration of 49 µM or 51 µM for each drug, and incubated for 24, 48, and 72 h. Moreover, HT29 cells were seeded onto 6-well plates, and after 24 h, they were treated with **(*R*)**-**5** at the concentration of 57 µM or with **(*S*)**-**5** at the concentration of 43 µM for 24, 48 and 72 h. The Trypan Blue exclusion dye method was used to determine the number of viable cells.

#### 3.2.1. MTT Assay

To evaluate **(*R*)**-**1, (*S*)**-**1, (*R*)**-**5** and **(*S*)**-**5** activity, the cells were treated for 24 h without (control) or with concentrations between 1 nM–5 mM (1 nM, 5 nM, 10 nM, 50 nM, 100 nM, 500 nM, 1 µM, 5 µM, 10 µM, 50 µM, 100 µM, 500 µM, 1 mM and 5mM) of the test samples. The culture medium was removed and the cells were further incubated for 2 h with 0.2 mg/mL MTT in PBS. After removal of the medium, the cells were lysed with 0.1 mL of iso-propanol. The absorbance of the solubilized formazan pellet at 540 nm was determined by Victor2, Multilabel plate reader (Perkin Elmer, MI, Italy). The IC_50_ was determined from three different experiments of the dose-response curve by using GraphPadPrism 5 (GraphPad Software, Inc., La Jolla, CA, USA) fitting a symmetrical sigmoidal shaped curve.

#### 3.2.2. Cell Cycle Analysis by Flow Cytometry

The HT29 cells were seeded in 25 cm^2^ flasks at a density of 2 × 10^4^ cells/cm^2^. The effects on the cell cycle were studied 48 h after treatment with 49 µM (*R*)-**1**. To determine the cell cycle distribution at the end of incubation, HT29 cells were stained according to Busi et al. [23]. Briefly, 1 × 10^6^ cells were pelleted and resuspended in trisodium citrate 0.1%, RNAse 0.1 mg/L, Igepal 0.01%, Propidium Iodide (PI) 50 µg/L. After 30 min at 37 °C in the dark, the cells were analyzed on a Coulter Epics Elite flow cytometer (Beckman Coulter) equipped with an argon ion laser tuned at 488 nm. PI fluorescence was collected on a linear scale at 600 nm and the DNA distribution was analyzed by the Modfit 5.0 software (ModFit Verity Software House http://www.vsh.com/). 

#### 3.2.3. Histone Extraction and Western Blot

HT29 cells were cultured with compounds (***R*)-1** for 6 h, and the histone fraction was extracted. The cells were harvested using 0.11% trypsin and 0.02% EDTA, washed twice with PBS, and nuclei were isolated according to Amellem et al. [24]. The nuclear histones were extracted, and proteins were quantified using a protein assay kit (Bio-Rad, Hercules, CA, USA). The histones were examined by 15% SDS-PAGE and a western blot analysis against acetylated lysines using anti-acetylated lysine (Cell Signaling Technology, Danvers, MA #9441, USA). The detection of the immunoreactive bands was performed with a secondary antibody conjugated with horseradish peroxidase (Amersham, Uppsala, SE NA931V, Sweden) and developed with the enhanced chemiluminescence system Clarity Western (Bio-Rad, Hercules, CA, USA), and the quantification was done by Fluor-S Max MultiImager (Bio-Rad) using the histone H3 signal as the control (Cell Signaling Technology, Beverly, MA #3638, USA).

#### 3.2.4. Total Protein Extraction and Western Blot

HT29 cells were treated with 49 µM of (***R*)-1** for 24 h. Cell were lysed in according to ref. 23. In brief, the cells were lysed for 20 min in an HEPES buffer and the protein concentration was determined by using the Bio-Rad protein assay method (Bio-Rad, Hercules, CA, USA). The proteins were resolved on a 10% density gel and immunoblotted with p21 (Cell Signaling Technology, Danvers, MA#2946, USA) or Myc (Cell Signaling Technology, Danvers, MA #5605, USA) or β-actin (Cell Signaling Technology, Danvers, MA #4967, USA) antibodies. The nitrocellulose membrane was incubated with secondary horseradish peroxidase-conjugated antibodies (GE Healthcare, Chicago, IL #NA931V or #NA9340, USA), developed as described in the previous section, and the quantification was done by Fluor-S Max MultiImager (Bio-Rad) using the β-actin signal as the control.

#### 3.2.5. Quantitative Real Time-PCR Analysis

HT29 cells were seeded in 25 cm^2^ flasks at a density of 2 × 10^4^ cells/cm^2^ and treated with **(*R*)-1** for 6 h. RT-PCR was assessed as previously described [23]. The gene expression was quantified by ΔΔC_T_ method, by using GAPDH as the housekeeping gene. The following primers were used: 5′-ATTTGGTCGTATTGGGCGCC-3′ (forward) and 5′-ACGGTGCCATGGAATTTGCC-3′ (reverse) for GAPDH detection, 5′-CCTAAGAGTGCTGGGCATTTT-3′ (forward) and 5′-TGAATTTCATAACCGCCTGTG-3′ (reverse) for P21 detection, 5′-TAGCTTCACCAACAGGAACT-3′ (forward) and 5′-AGCTCGAATTTCTTCCAGAT-3′ (reverse) for MYC detection.

### 3.3. Statistical Analysis

The data for the MTT, western blot, cell cycle and RT-PCR were analysed with a one-way analysis of variance (ANOVA) followed by Dunnett’s multiple comparison test. The data of the cell growth were analysed with a two-way analysis of variance (ANOVA) followed by the Bonferroni multiple comparisons. The differences of at least *p* < 0.05 were considered significant. A statistical analysis was carried out using Prism GraphPad software.

## 4. Conclusions

The novel derivatives of (*9R*)-9-hydroxystearic acid (9-HSA) have been synthesized and the influence of the modification at the level of the substituents on C-9 and of the carboxy group have been studied on HT 29 colon cancer cell line.

The modification on the C-9 by ester (namely methyl (9*R*)-9-{[(4-methylphenyl)sulphonyl]oxy}octadecanoate, **(*R*)**-**2**), by ether (namely methyl 9-methoxyoctadecanoate, **3**), or azide group did not produce relevant antiproliferative activity on HT 29 cells. On the contrary, methyl (*9S*)-9-aminooctadecanoate [**(*S*)**-**5**] and its enantiomeric form **(*R*)-5** was active against the above colon tumor cells, but very toxic, as evidenced by the high cytotoxicity after 48 and 72 h of the treatment. This is an indication of the importance, for a biological effect, to have a group bound to the C-9 with hydrogen atoms able to interact with the active site of the biological target. When the modification was at the carboxy group of 9-HSA, as in case of the formation of methyl esters **(*R*)**-**1** and **(*S*)**-**1**, an antiproliferative effect was observed. In particular, **(*R*)**-**1** showed a more marked cytostatic effect compared to the **(*S*)**-**1** enantiomer. In fact, the number of cells dropped at 24 h and remained unchanged over time, suggesting a halt in cell growth. The anti-proliferative effect induced by **(*R*)**-**1** is characterized by an accumulation of cells in the G0 / G1 phase. The antiproliferative effect of the **(*R*)**-**1** is due to an increase of the p21WAF/CYP1 and a decrease of c-myc expression that is reflected on these two protein levels.

## Supplementary Materials

The following are available online: Figures S1–S18: ^1^H and ^13^C-NMR spectra of compounds **1**–**8**.

## References

[1] Masotti L., Casali E., Gesmundo N., Sartor G., Galeotti T., Borrello S., Piretti M.V., Pagliuca G. (1989). Lipid peroxidation in cancer cells: Chemical and physical studies. Ann. N. Y. Acad. Sci..

[2] Masotti L., Casali E., Gesmundo N. (1993). Influence of hydroxystearic acid on in vitro cell proliferation. Mol. Aspects Med..

[3] Bertucci C., Hudaib M., Boga C., Calonghi N., Cappadone C., Masotti L. (2002). Gas chromatography/ mass apectrometry assay of endogenous cellular lipid peroxidation products: Quantitative analysis of 9- and 10-hydroxystearic acids. Rapid Commun. Mass Spectrom..

[4] Calonghi N., Cappadone C., Pagnotta E., Farruggia G., Buontempo F., Boga C., Brusa G.L., Santucci M.A., Masotti L. (2004). 9-Hydroxystearic acid upregulates p21WAF1 in HT29 cancer cells. Biochem. Biophys. Res. Commun..

[5] Calonghi N., Pagnotta E., Parolin C., Molinari C., Boga C., Dal Piaz F., Brusa G.L., Santucci M.A., Masotti L. (2007). Modulation of apoptotic signalling by 9-hydroxystearic acid in osteosarcoma cells. Biochim. Biophys. Acta, Mol. Cell. Biol. Lipids.

[6] Boanini E., Torricelli P., Boga C., Micheletti G., Cassani M.C., Fini M., Bigi A. (2016). (9R)-9-Hydroxystearate-Functionalized Hydroxyapatite as Anti-Proliferative and Cytotoxic Agent towards Osteosarcoma Cells. Langmuir.

[7] Calonghi N., Cappadone C., Pagnotta E., Boga C., Bertucci C., Fiori J., Tasco G., Casadio R., Masotti L. (2005). Histone deacetylase 1: A target of 9-hydroxystearic acid in the inhibition of cell growth in human colon cancer. J. Lipid Res..

[8] Calonghi N., Pagnotta E., Parolin C., Tognoli C., Boga C., Masotti L. (2006). 9-Hydroxystearic acid interferes with EGF signalling in a human colon adenocarcinoma. Biochem. Biophys. Res. Commun..

[9] West A.C., Johnstone R.W. (2014). New and emerging HDAC inhibitors for cancer treatment. J. Clin. Invest..

[10] Earle F.R., Mikolajczak K.L., Wolff I.A., Barclay A.S. (1964). Search for new industrial oils. X. Seed oils of the calenduleae. J. Am. Oil Chem. Soc..

[11] Parolin C., Calonghi N., Presta E., Boga C., Caruana P., Naldi M., Andrisano V., Masotti L., Sartor G. (2012). Mechanism and stereoselectivity of HDAC I inhibition by (*R*)-9-hydroxystearic acid in colon cancer. Biochim. Biophys. Acta.

[12] Ebert C., Felluga F., Forzato C., Foscato M., Gardossi L., Nitti P., Pitacco G., Boga C., Caruana P., Micheletti G. (2012). Enzymatic kinetic resolution of hydroxystearic acids: A combined experimental and molecular modelling investigation. J. Molec. Catal. B: Enzymatic.

[13] Bayley H., Standring D.N., Knowles J.R. (1978). Propane-1,3-di-thiol: A selective reagent for the efficient reduction of alkyl and aryl azides to amines. Tetrahedron Lett..

[14] Boga C., Drioli S., Forzato C., Micheletti G., Nitti P., Prati F. (2016). Easy route to enantiomerically enriched 7- and 8-hydroxy-stearic acids by olefin-metathesis-based approach. Synlett.

[15] Richon V.M., Sandhoff T.W., Rifkind R.A., Marks P.A. (2000). Histone deacetylase inhibitor selectively induces p21WAF1 expression and gene-associated histone acetylation. Proc. Natl. Acad. Sci. USA.

[16] Nakano K., Mizuno T., Sowa Y., Orita T., Yoshino T., Okuyama Y., Fujita T., Ohtani-Fujita N., Matsukawa Y., Tokino T. (1997). Butyrate activates the WAF1/Cip1 gene promoter through Sp1 sites in a p53-negative human colon cancer cell line. J. Biol. Chem..

[17] Huang L., Sowa Y., Sakai T., Pardee A.B. (2000). Activation of the p21WAF1/CIP1 promoter independent of p53 by the histone deacetylase inhibitor suberoylanilide hydroxamic acid (SAHA) through the Sp1 sites. Oncogene.

[18] Dang C.V. (2013). MYC, metabolism, cell growth, and tumorigenesis. Cold Spring Harb. Perspect. Med..

[19] Lawlor E.R., Soucek L., Brown-Swigart L., Shchors K., Bialucha C.U., Evan G.I. (2006). Reversible kinetic analysis of Myc targets in vivo provides novel insights into Myc-mediated tumorigenesis. Cancer Res..

[20] Sodir N.M., Swigart L.B., Karnezis A.N., Hanahan D., Evan G.I., Soucek L. (2011). Endogenous Myc maintains the tumor microenvironment. Genes Dev..

[21] Wolfer A., Wittner B.S., Irimia D., Flavin R.J., Lupien M., Gunawardane R.N., Meyer C.A., Lightcap E.S., Tamayo P., Mesirov J.P. (2010). MYC regulation of a “poor-prognosis” metastatic cancer cell state. Proc. Natl. Acad. Sci. USA.

[22] Cochrane C., Harwood H.J. (1961). Phase properties of mixtures of 9- and 10-oxo-octadecanoic acids and of 9- and 10-hydroxyoctadecanoic acids. J. Org. Chem..

[23] Busi A., Aluigi A., Guerrini A., Boga C., Sartor G., Calonghi N., Sotgiu G., Posati T., Corticelli F., Fiori J. (2019). Unprecedented behavior of (9R)-9-hydroxystearic acid loaded keratin nanoparticles on cancer cell cycle. Mol. Pharmaceutics.

[24] Amellem O., Stokke T., Sandvik J.A., Pettersen E.O. (1996). The retinoblastoma gene product is reversibly dephosphorylated and bound in the nucleus in S and G2 phases during hypoxic stress. Exp. Cell Res..

