# Supplementary material for: Synthesis of 9-Hydroxystearic Acid Derivatives and Their Antiproliferative Activity on HT 29 Cancer Cells
Source: Molecules. 2019 Oct 15;24(20):3714. doi: 10.3390/molecules24203714 (PMC6832665; doi:10.3390/molecules24203714)
Supplement: Supplementary file 1 [file molecules-24-03714-s001.pdf]

Synthesis of 9-hydroxystearic acid derivatives and their antiproliferative activity on HT 29 cancer cells

Natalia Calonghi[1]*, Carla Boga[2]*, Dario Telese[2], Silvia Bordoni[2], Giorgio Sartor[1], Chiara Torsello[1], and Gabriele Micheletti[2]

1 Department of Pharmacy and Biotechnology, University of Bologna, Bologna (Italy); (NC), giorgio.sartor@unibo.it (GS), chiara.torsello@virgilio.it (CT)

2 Department of Industrial Chemistry 'Toso Montanari', Alma Mater Studiorum Università di Bologna Viale Del Risorgimento, 4 402136 Bologna Italy; (CB), dario.telese2@unibo.it (DT), silvia.bordoni@unibo.it (SB), gabriele.micheletti3@unibo.it (GM)

Supporting information

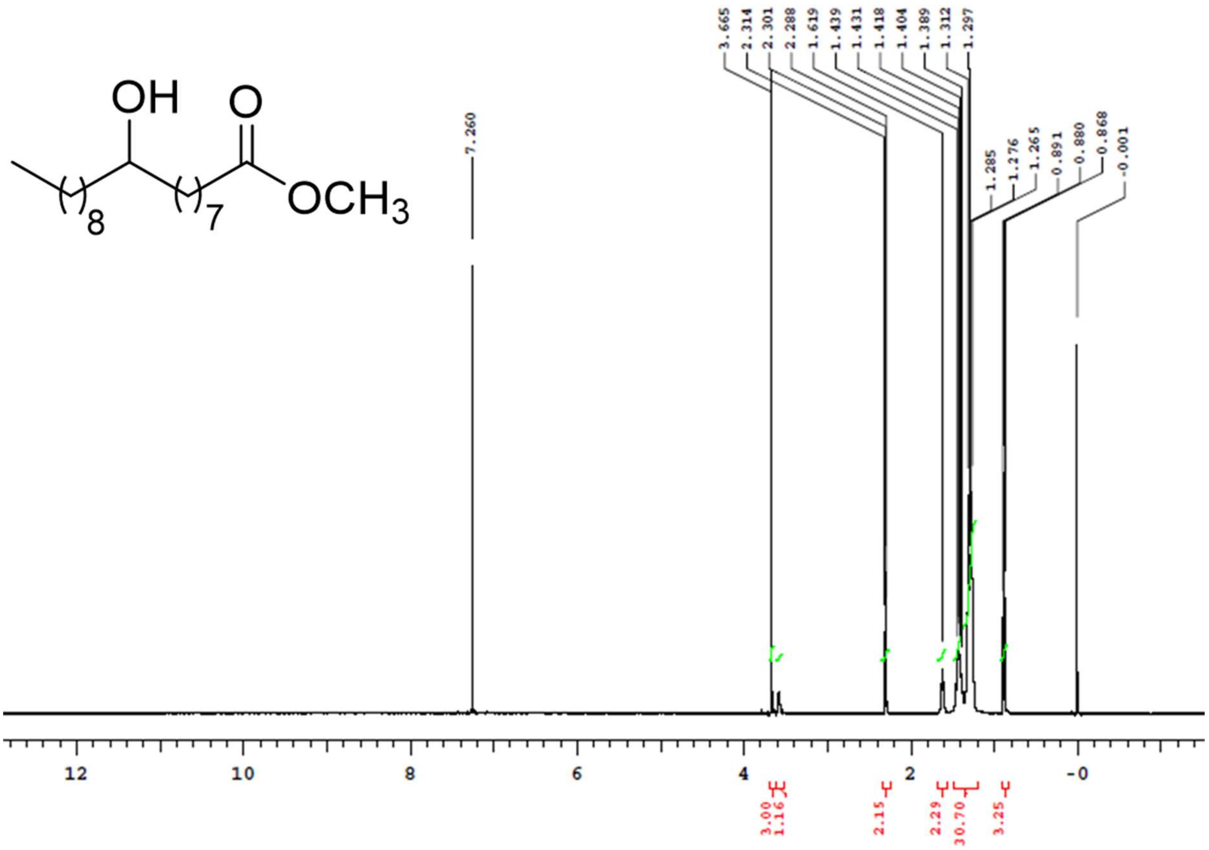

Figure SI-1. ^{1}H NMR spectrum (600 MHz, $CDCl_3$) of compound **1**.

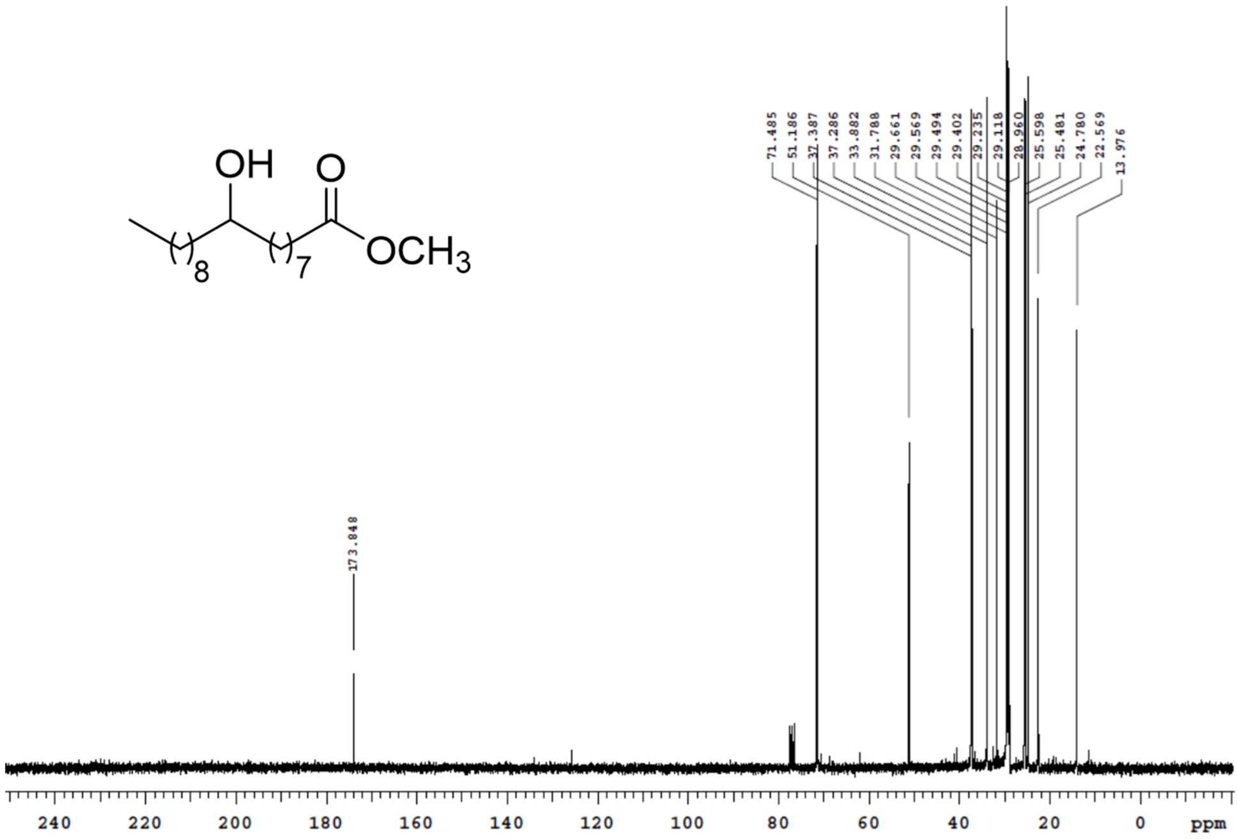

Figure SI-2. ^{13}C NMR spectrum (400 MHz, $CDCl_3$) of compound **1**.

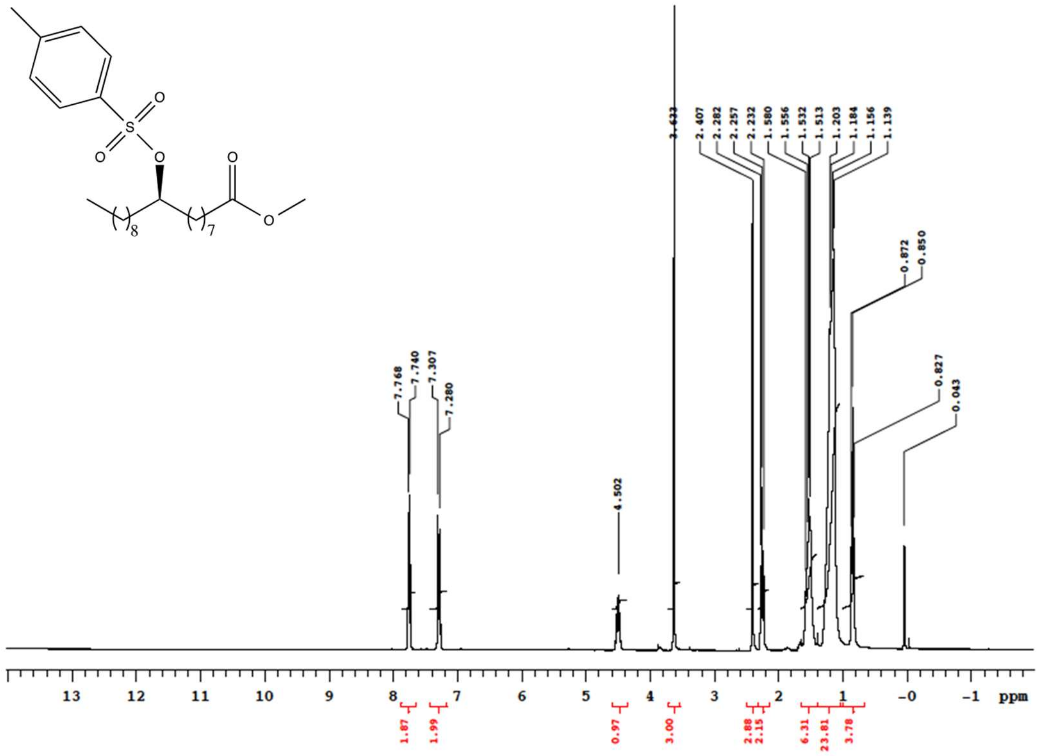

Figure SI-3. ^{1}H NMR spectrum (300 MHz, $CDCl_3$) of compound **(*R*)-2**.

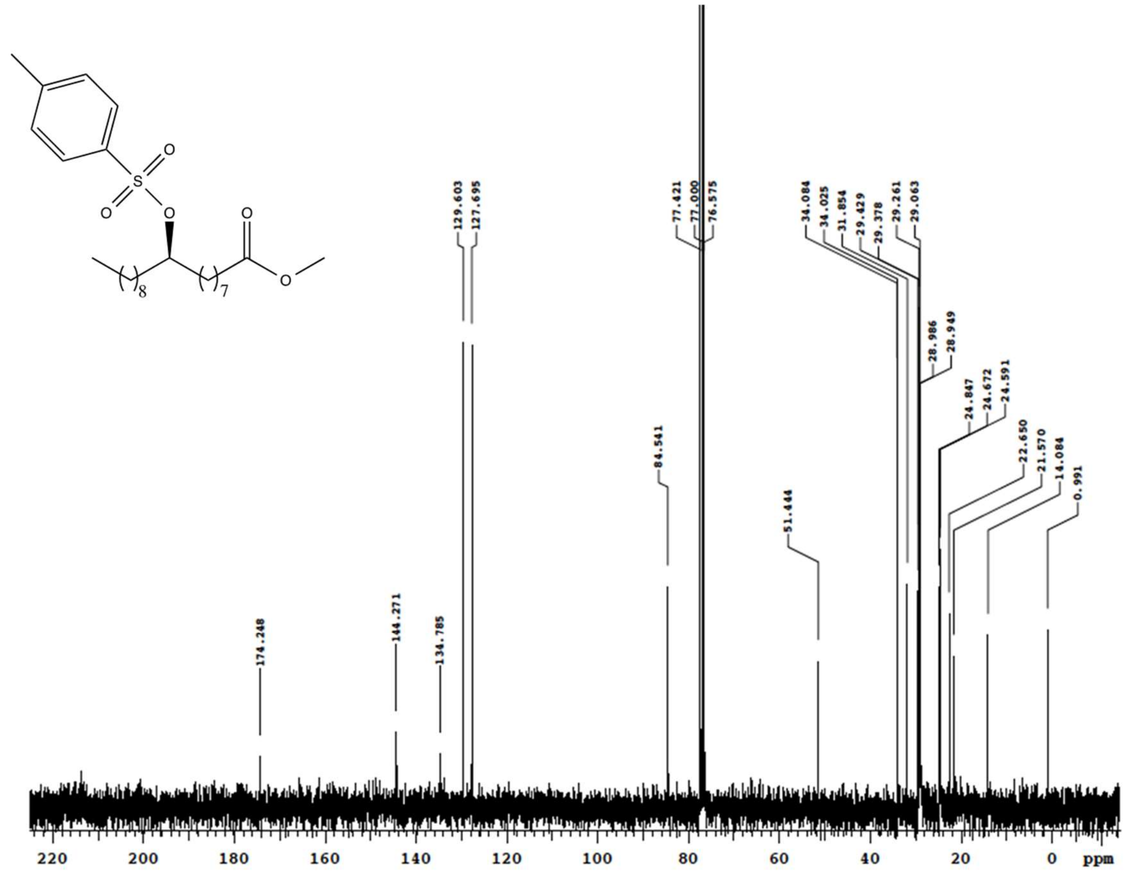

Figure SI-4. ^{13}C NMR spectrum (75.44 MHz, $CDCl_3$) of compound **(*R*)-2**.

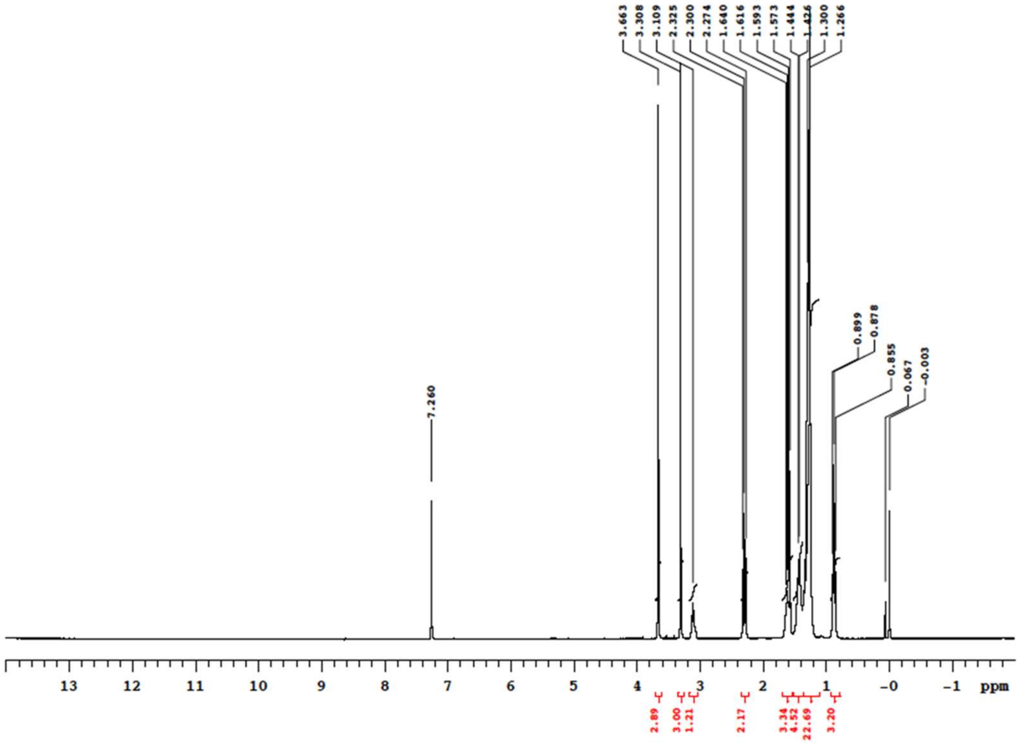

Figure SI-5. ^{1}H NMR spectrum (300 MHz, $CDCl_3$) of compound **3**.

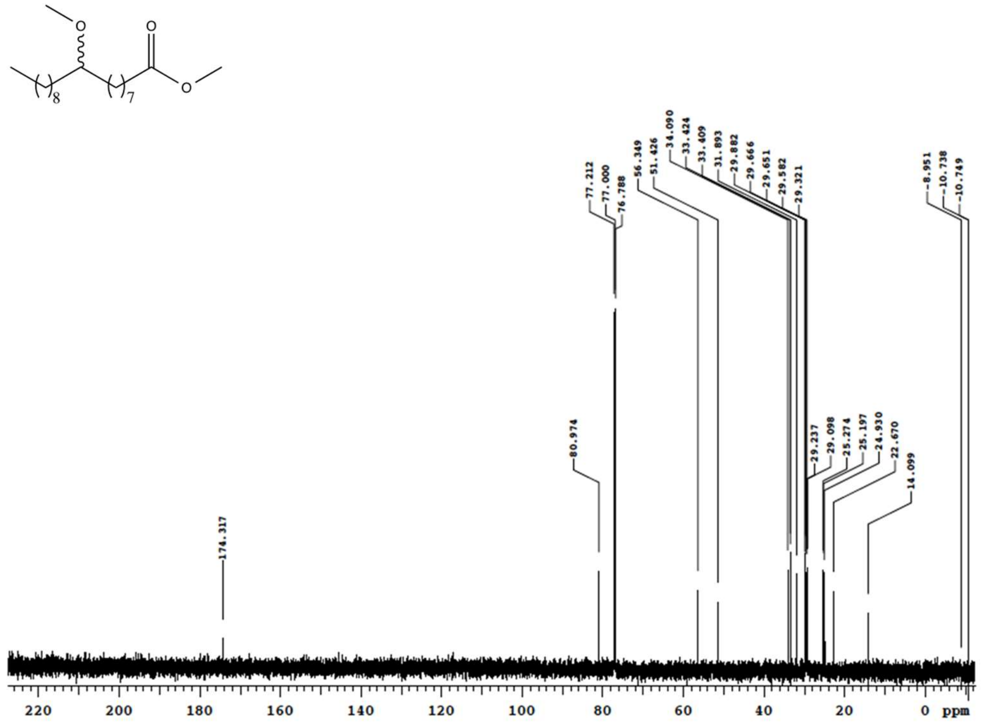

Figure SI-6. ^{13}C NMR spectrum (150.80 MHz, $CDCl_3$) of compound **3**.

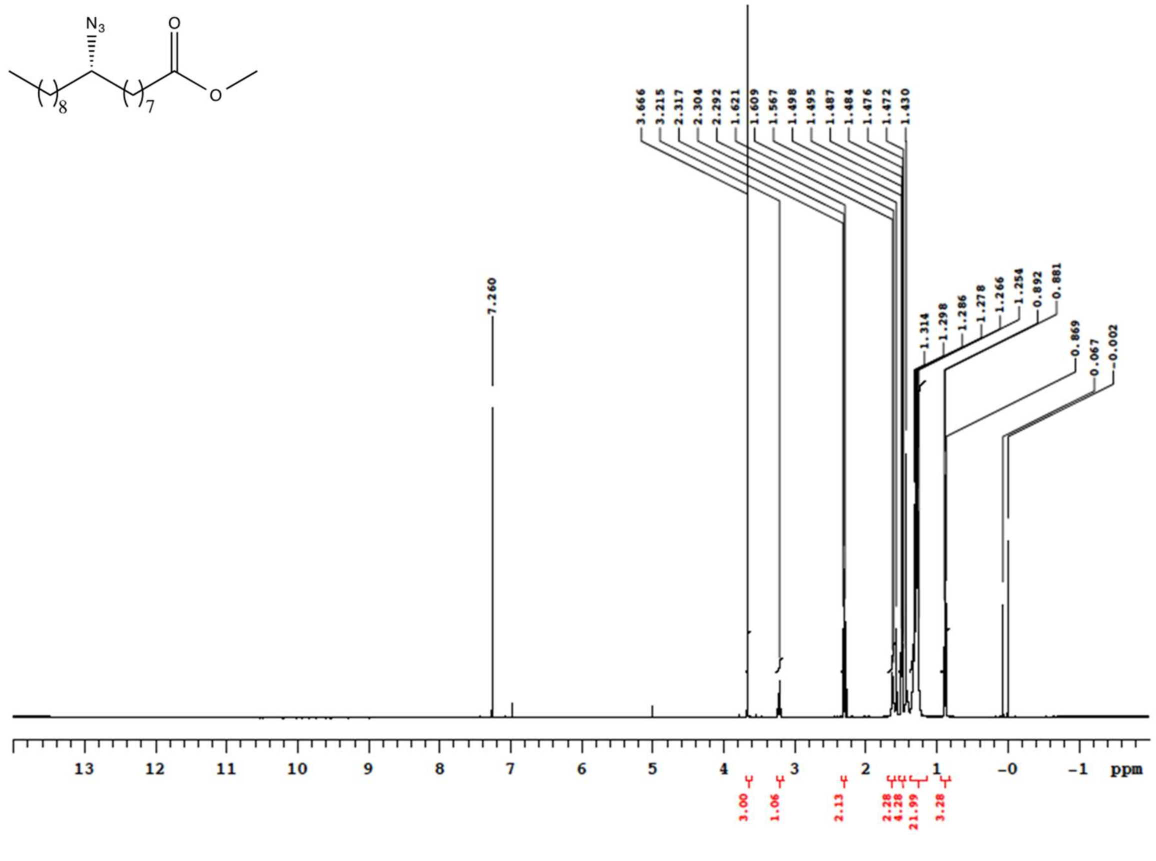

Figure SI-7. ^{1}H NMR spectrum (300 MHz, $CDCl_3$) of compound **(*S*)-4**.

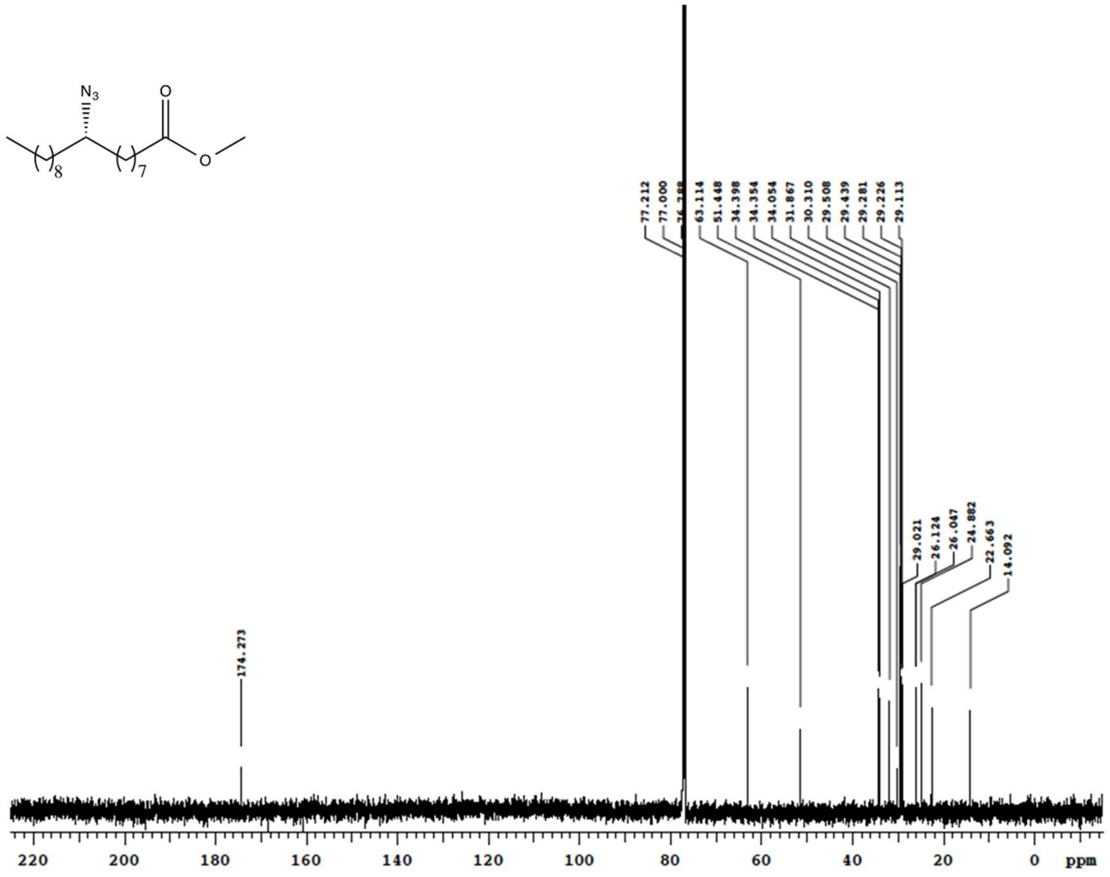

Figure SI-8. ^{13}C NMR spectrum (150.80 MHz, $CDCl_3$) of compound **(S)-4**.

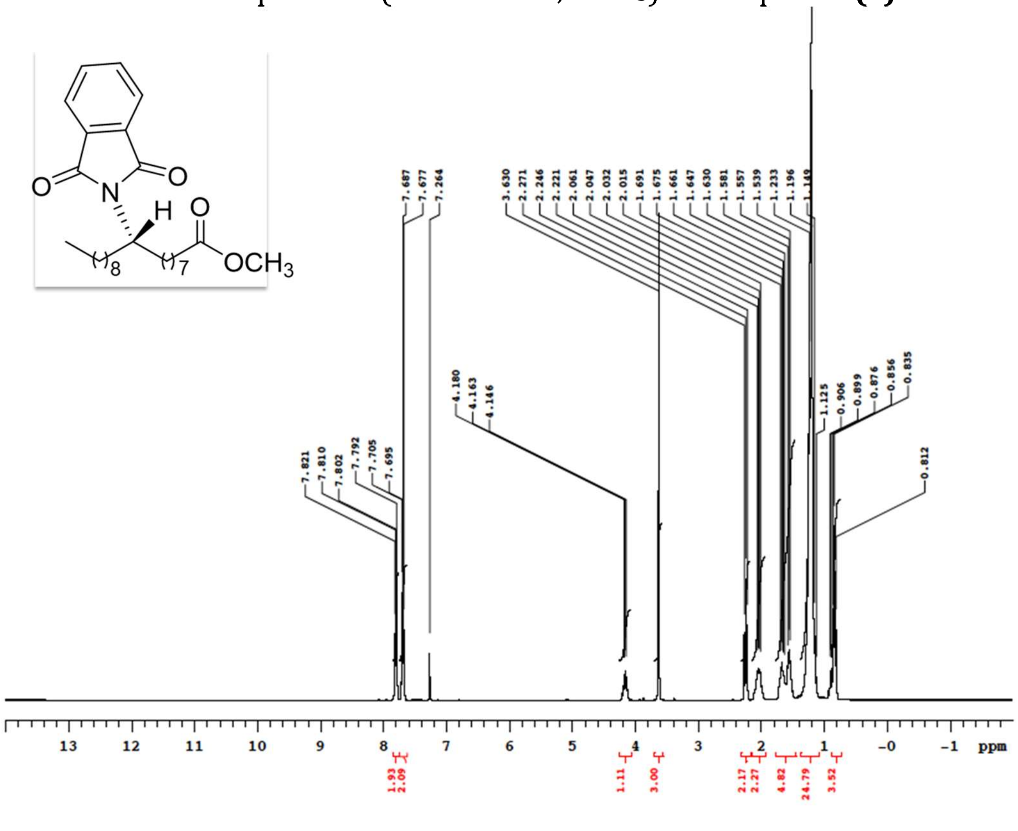

Figure SI-9. ^{1}H NMR spectrum (400 MHz, $CDCl_3$) of compound **(S)-6**.

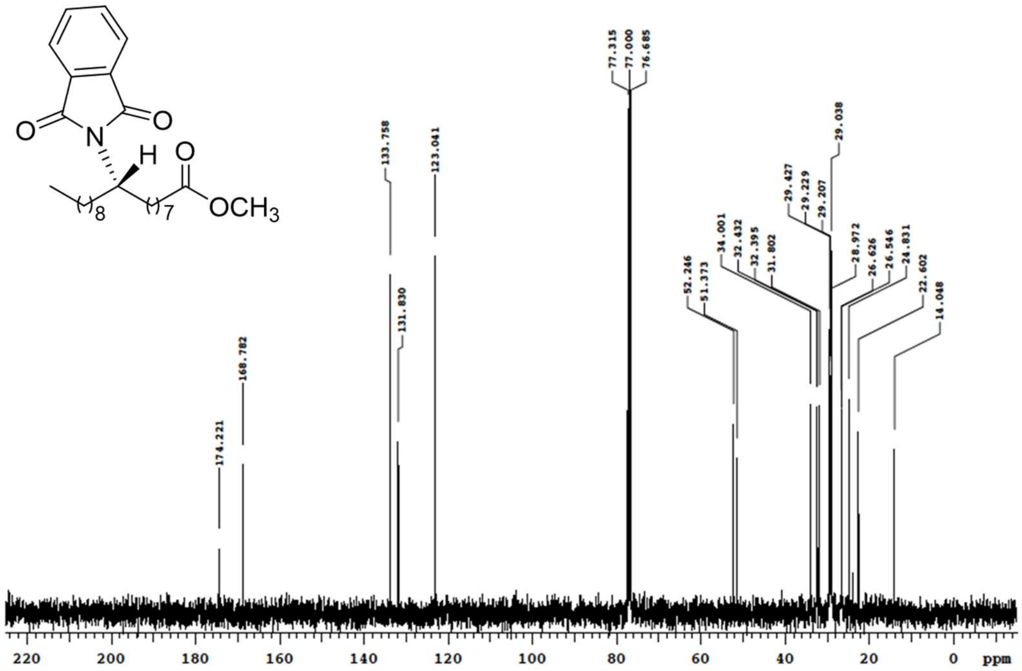

Figure SI-10. ^{13}C NMR spectrum (100.56 MHz, $CDCl_3$) of compound ***(S)*-6**.

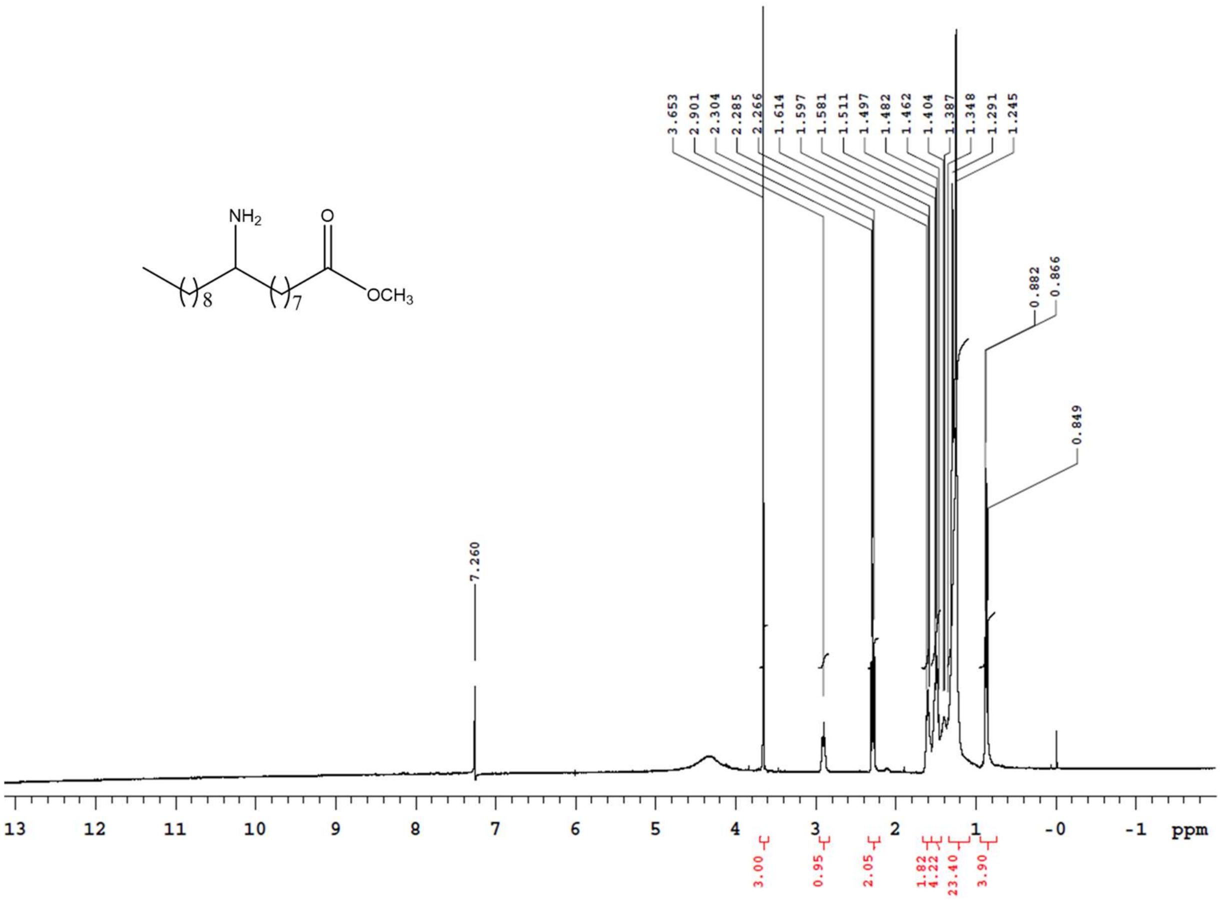

Figure SI-11. ^{1}H NMR spectrum (400 MHz, $CDCl_3$) of compound **5**.

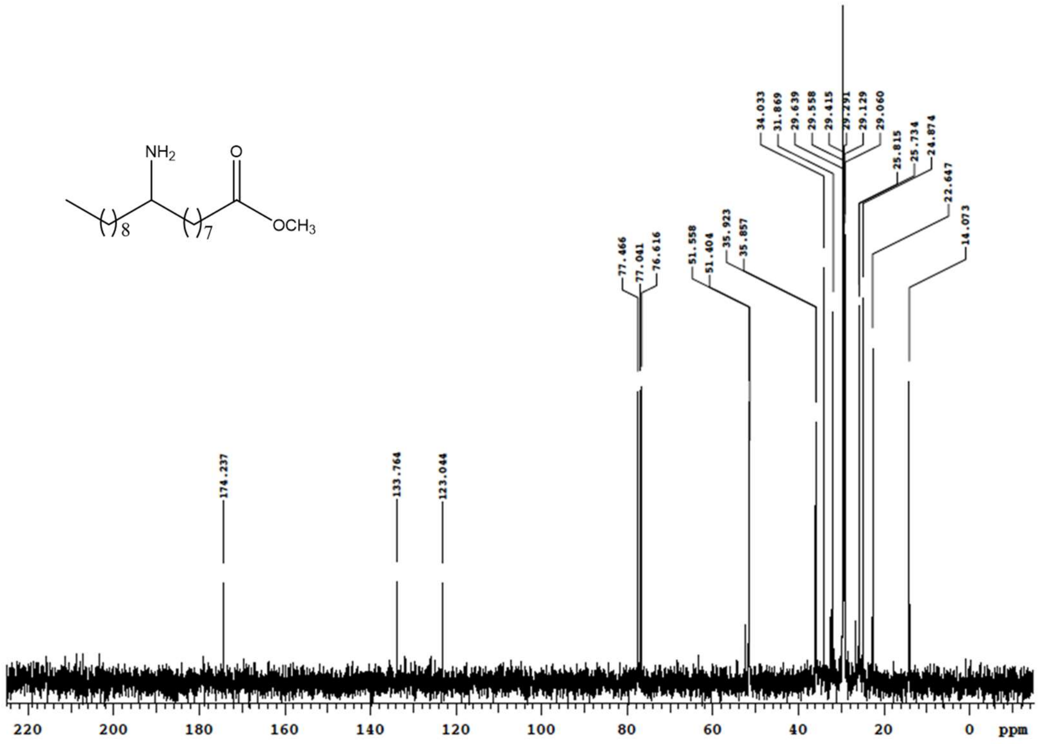

Figure SI-12. ^{13}C NMR spectrum (100.56 MHz, $CDCl_3$) of compound **5**.

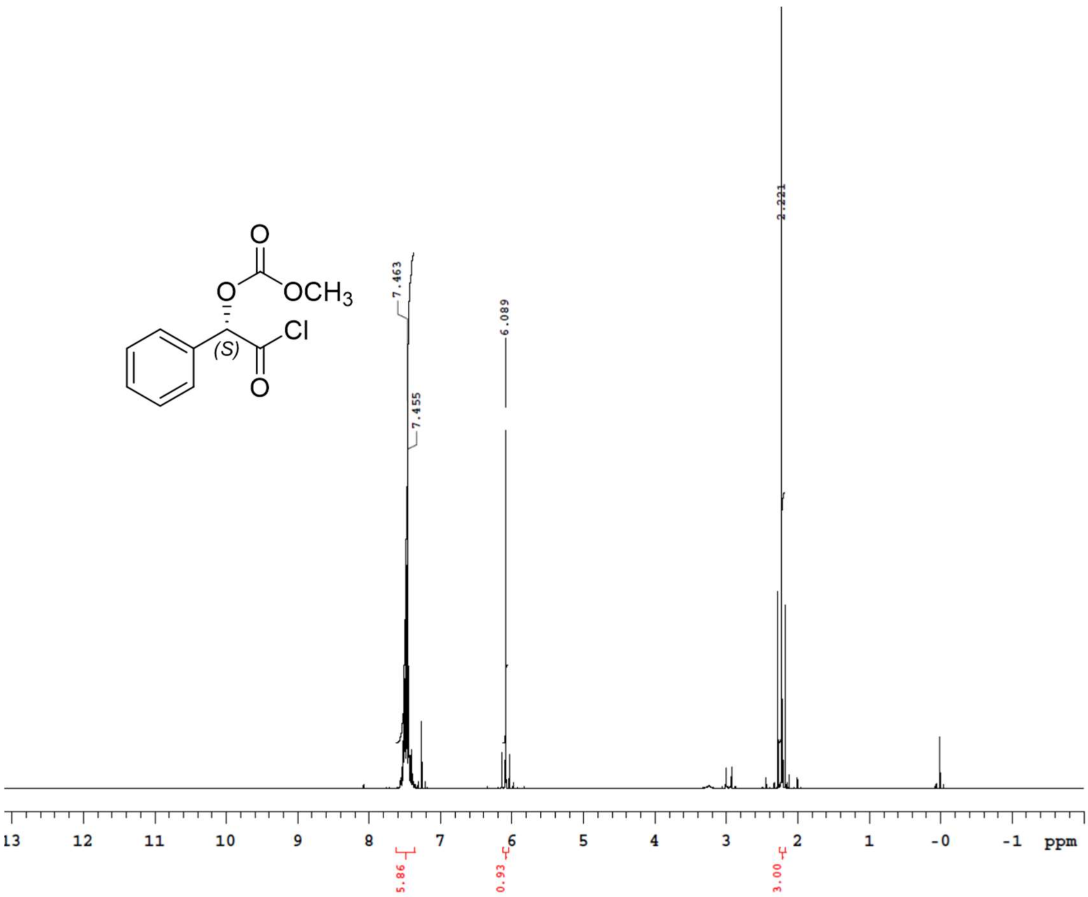

Figure SI-13. ^1H NMR spectrum (300 MHz, $CDCl_3$) of compound **7**.

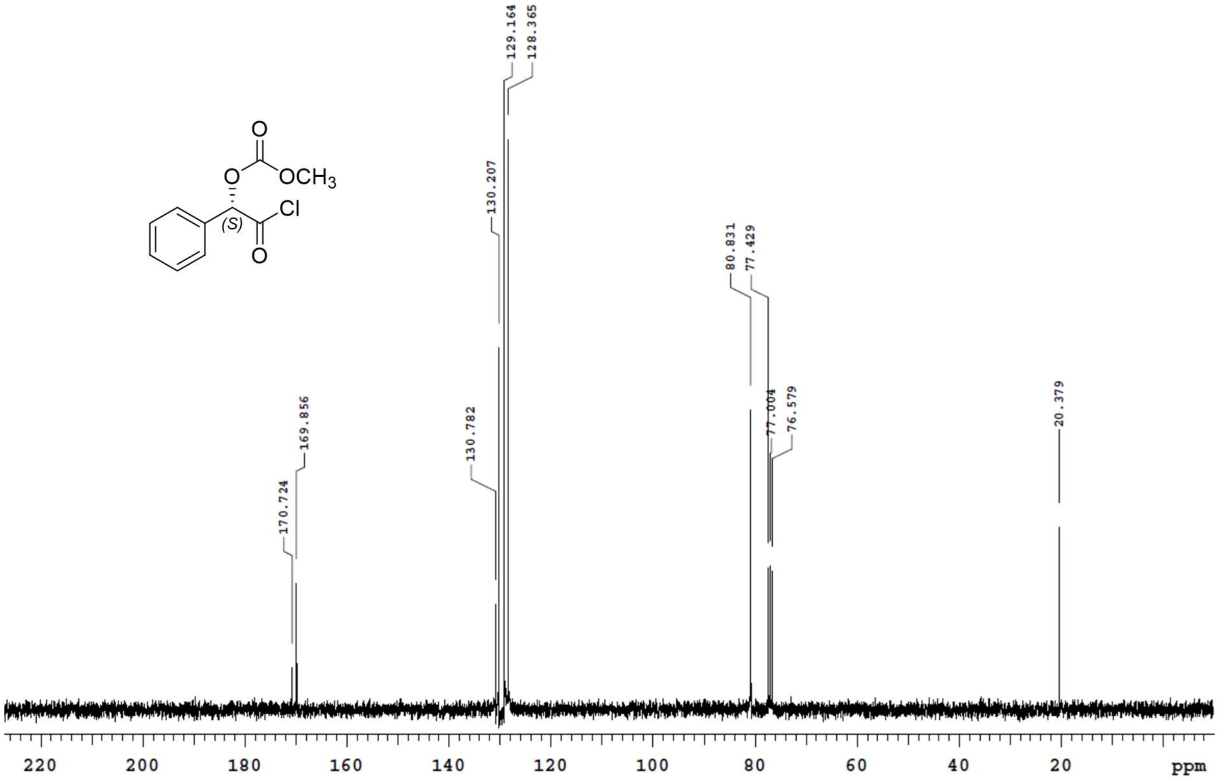

Figure SI-14. ^{13}C NMR spectrum (75.44 MHz, $CDCl_3$) of compound **7**.

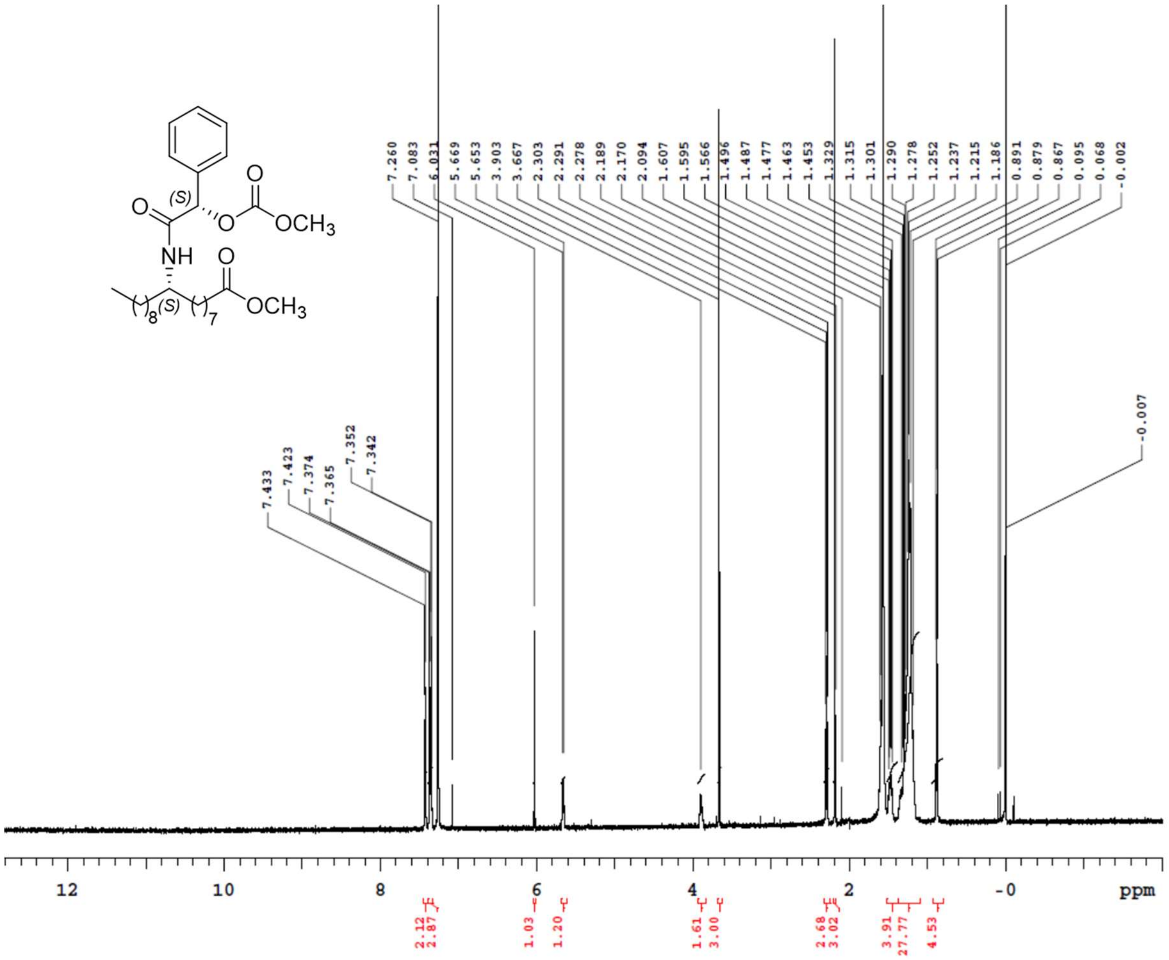

Figure SI-15. ^{1}H NMR spectrum (600 MHz, $CDCl_3$) of compound ***(S,S)*-8**

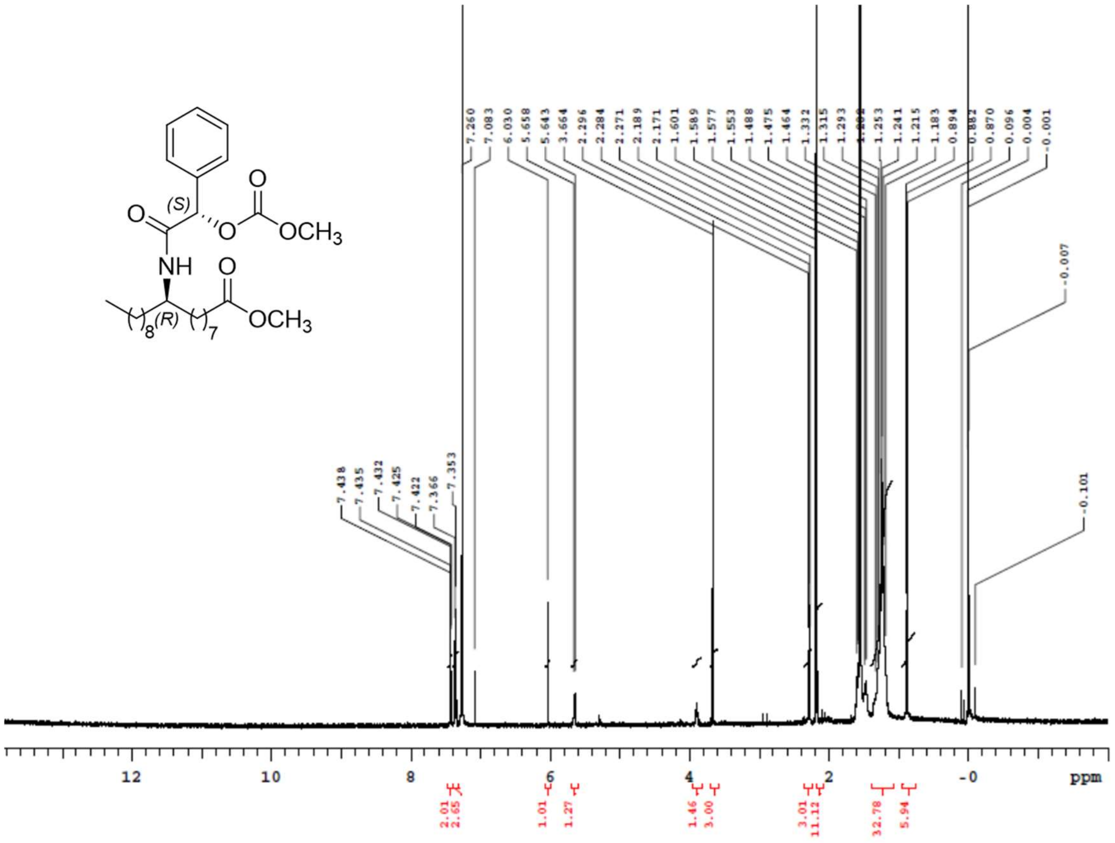

Figure SI-16. ^{1}H NMR spectrum (600 MHz, $CDCl_3$) of compound ***(R,S)*-8**

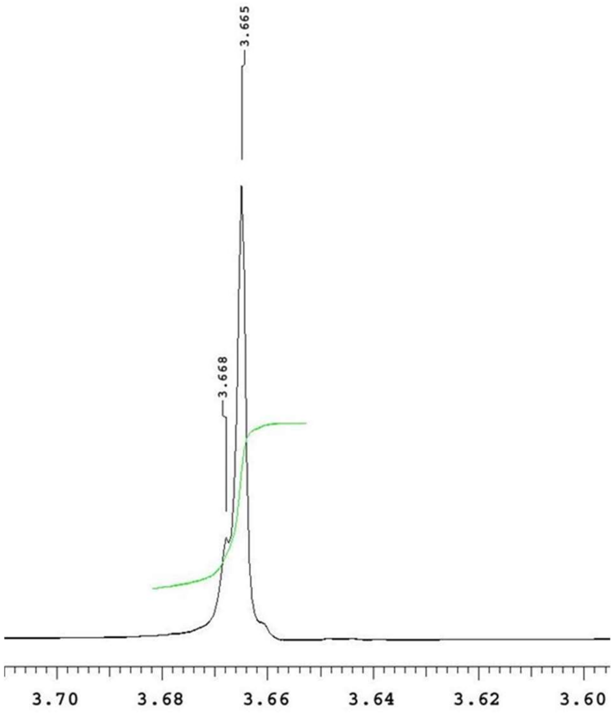

Figure SI-17 Expanded view around the signal belonging to methoxy hydrogen atoms in the ^{1}H NMR spectrum (600 MHz, $CDCl_3$) of the crude reaction mixture obtaining by reacting **(*R*)-5**-with **7**.

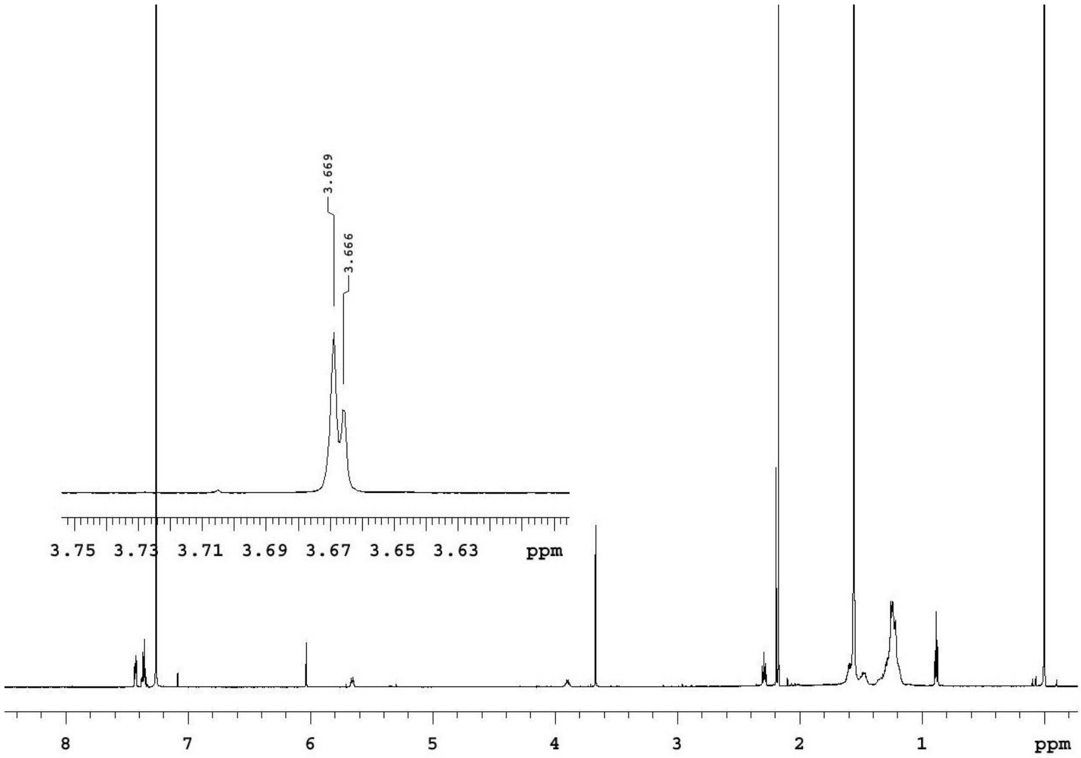

Figure SI-18. ^{1}H NMR spectrum in $CDCl_3$ (600 MHz) obtained by mixing different amount of ***(S,S)*-8** and ***(R,S)*-8** previously purified by preparative TLC on silica gel 20x20 glass plate. The presence of two distinct signals at 3.669 ppm and 3.666 ppm confirms that the signal of the two diastereoisomers can be separated. Up: expanded view of the methoxyl signals region.
